# Differential gene expression profiling of human bone marrow-derived mesenchymal stem cells during adipogenic development

**DOI:** 10.1186/1471-2164-12-461

**Published:** 2011-09-24

**Authors:** Adriane Menssen, Thomas Häupl, Michael Sittinger, Bruno Delorme, Pierre Charbord, Jochen Ringe

**Affiliations:** 1 Tissue Engineering Laboratory, Clinic for Rheumatology and Clinical Immunology, Charité-Universitätsmedizin Berlin, Charité Platz 1, 10117 Berlin, Germany; 2 Berlin-Brandenburg Center for Regenerative Therapies, Charité-Universitätsmedizin Berlin, Föhrer Str. 15, 13353 Berlin, Germany; 3 MacoPharma, Tourcoing, France; 4 INSERM U972, University Paris 11, Le Kremlin-Bicêtre, France; 5 Leibniz Institute for Molecular Pharmacology (FMP), Campus Berlin-Buch, Robert-Rössle-Str.10, 13125 Berlin, Germany

## Abstract

**Background:**

Adipogenesis is the developmental process by which mesenchymal stem cells (MSC) differentiate into pre-adipocytes and adipocytes. The aim of the study was to analyze the developmental strategies of human bone marrow MSC developing into adipocytes over a defined time scale. Here we were particularly interested in differentially expressed transcription factors and biochemical pathways. We studied genome-wide gene expression profiling of human MSC based on an adipogenic differentiation experiment with five different time points (day 0, 1, 3, 7 and 17), which was designed and performed in reference to human fat tissue. For data processing and selection of adipogenic candidate genes, we used the online database SiPaGene for Affymetrix microarray expression data.

**Results:**

The mesenchymal stem cell character of human MSC cultures was proven by cell morphology, by flow cytometry analysis and by the ability of the cells to develop into the osteo-, chondro- and adipogenic lineage. Moreover we were able to detect 184 adipogenic candidate genes (85 with increased, 99 with decreased expression) that were differentially expressed during adipogenic development of MSC and/or between MSC and fat tissue in a highly significant way (p < 0.00001). Subsequently, groups of up- or down-regulated genes were formed and analyzed with biochemical and cluster tools. Among the 184 genes, we identified already known transcription factors such as *PPARG*, *C/EBPA *and *RTXA*. Several of the genes could be linked to corresponding biochemical pathways like the adipocyte differentiation, adipocytokine signalling, and lipogenesis pathways. We also identified new candidate genes possibly related to adipogenesis, such as *SCARA5*, coding for a receptor with a putative transmembrane domain and a collagen-like domain, and *MRAP*, encoding an endoplasmatic reticulum protein.

**Conclusions:**

Comparing differential gene expression profiles of human MSC and native fat cells or tissue allowed us to establish a comprehensive differential kinetic gene expression network of adipogenesis. Based on this, we identified known and unknown genes and biochemical pathways that may be relevant for adipogenic differentiation. Our results encourage further and more focused studies on the functional relevance of particular adipogenic candidate genes.

## Background

Human mesenchymal stem cells (MSC) are easy to isolate from bone marrow aspirates. In cell culture, they can be expanded as clones showing multilineage differentiation potential [1,2]. It is well known that human MSC differentiate when cultured under appropriate conditions into adipocytes, osteoblasts or chondrocytes [1,3].

Human adipocyte development can be studied *in vitro *starting from MSC cultures, which can be induced to follow the process of adipogenesis [4]. How to grow MSC obtained from bone marrow aspirates and other tissues under adipogenic differentiation conditions [5,6] is already well established. Insulin is known to act through the insulin-like growth factor receptor 1. Dexamethasone, a synthetic glucocorticoid agonist is used to stimulate the glucocorticoid receptor pathway and methylisobutylxanthine, a cAMP-phosphodiesterase inhibitor, are used to raise the cAMP level and thus to stimulate the cAMP dependent protein kinase pathway. Here, we exposed cultured MSC to adipogenic conditions in order to examine their adipogenic differentiation potential by the observation of lipid droplets stained with oil red O.

In recent years, new cellular and molecular insights into adipogenesis have been obtained by combining MSC as an *in vitro *model for adipogenic differentiation and new "omics" technologies as monitoring tools. Transcriptomics in combination with bio-informatics were not only essential in providing a list of potential adipogenic key player genes, they also allowed for a preliminary global view on biological processes and molecular networks involved in adipogenesis [7-9], whereas proteomics of adipogenically differentiated MSC were important to verify transcriptomic data [10]. Furthermore, epigenomic approaches have allowed deeper insight in the epigenetic programming of MSC from human fat tissue [11], and state-of-the-art microRNA array technology revealed the influence of non-coding RNA on MSC adipogenesis [12,13]. In particular, miR-27a was found to be a negative regulator of adipogenesis via the suppression of *PPARG *expression [13].

During adipogenesis derived from MSC, the gene expression profile represents a unique, albeit not totally deciphered, pattern of transcription factors, allowing for the differentially induced regulation of specific pre-adipogenic genes to form pre-adipocytes. These regulatory factors promote further downstream target gene expression, responsible for the formation of typical adipocytes. *In vivo*, adipose tissue consists of, for one half, classical adipocytes, in which growth and long-term change and storage of lipids and fatty acids can be observed. The other half is composed of pre-adipocytes and supporting cells such as fibroblasts, immune cells (macrophages) or different blood cells [14]. It has been shown that adipocytes cannot grow and accumulate lipids without restriction: for example, after reaching a certain size they are forced to divide into two or more adipocytes. The two observed processes, called hypertrophy (increase in adipocyte size) and hyperplasia (increase in adipocyte number) are typical observations of adipose tissue expansion [15]. After initial expansion under standard culture conditions, cellular growth arrest of proliferating pre-adipocytes is stopped when reaching confluence. However, following the addition of a hormonal cocktail, the cells undergo further cell divisions. In most cases this phenomenon occurs before the terminal differentiation program starts, and development into mature adipocytes begins.

The overall goal of our study was to analyze the pattern of gene expression of human bone marrow MSC differentiation into adipocytes during a defined time scale. We were particularly interested in differentially expressed transcription factors corresponding to biochemical pathways involved in the process of adipogenesis. Therefore we report genome-wide gene expression profiling of three human MSC lines on adipogenic differentiation at five different time points (day 0, 1, 3, 7 and 17) in reference to normal human fat cell tissue. Thereafter, the expression profiles of all cells were analyzed using the online SiPaGene database for Affymetrix microarray expression data, which was set up for chip comparison experiments [16]. With this approach, it was possible to describe a specific pattern of gene expression during adipogenic differentiation of human MSC and between these MSC and native human fat tissue.

## Results

### Isolation, culture and surface marker presentation of human MSC

**Figure 1:**
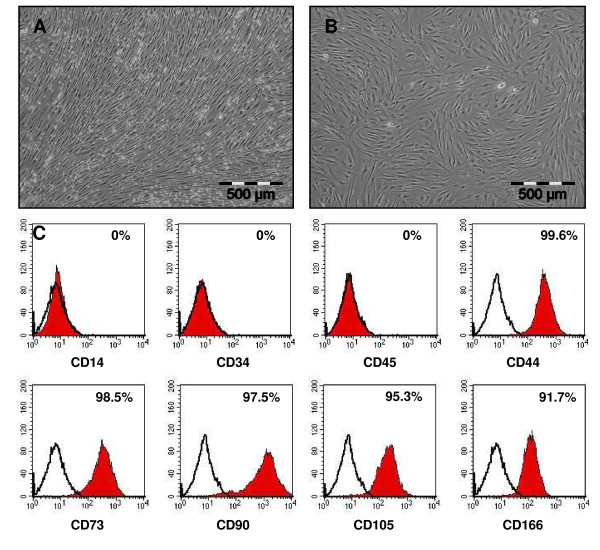
**Morphology and surface marker presentation of human MSC (A-C)**. Human mesenchymal stem cells (MSC) were isolated from bone marrow aspirates. During primary culture (A) and up to passage 3 (B) they showed a typical fibroblast-like morphology and grew in a swirling-like pattern. Flow cytometry analysis demonstrated a homogenous MSC population (C). As expected, MSC were negative for reactivity to antigens CD14, CD34 and CD45, but positive for reactivity to antigens CD44, CD73, CD90, CD105 and CD166.

Human MSC were isolated from bone marrow aspirates and expanded in standard culture media containing selected batches of fetal bovine serum and 1 ng/mL basic-FGF. Cultures showed the typical fibroblast-like cell morphology of MSC growing in a swirling pattern during primary culture (Figure 1-A) up to passage 3 (Figure 1-B). To verify cell identity, cells were routinely investigated using flow cytometry analysis. Measurements revealed a uniformly negative cell population for the hematopoietic markers CD14, CD34 and CD45 (Figure 1-C), and positive for CD44, CD73, CD90, CD105 and CD166 (Figure 1-C).

### Osteogenic, chondrogenic and adipogenic potential of MSC

**Figure 2:**
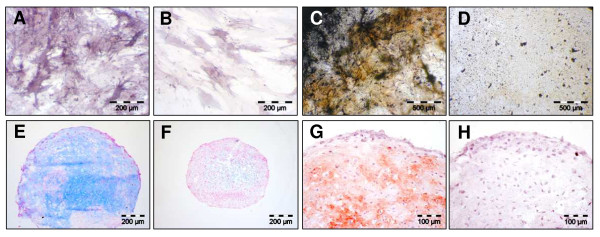
**Osteogenic and chondrogenic differentiation of human MSC (A-H)**. On day 21, alkaline phosphatase staining of osteogenically induced MSC cultures demonstrated an enhanced AP activity (A) when compared to control cultures (B). On this day, von Kossa staining of osteogenically induced cultures was positive indicating a formation of mineralized bone matrix (C), whereas control cultures stained negative (D). TGFβ3 - induction of chondrogenesis, on day 21, resulted in the secretion of cartilage proteoglycans as shown by alcian blue staining (E), and of cartilage specific collagen type II (G). Control cultures showed no secretion of both extracellular matrix molecules (F, H).

**Figure 3:**
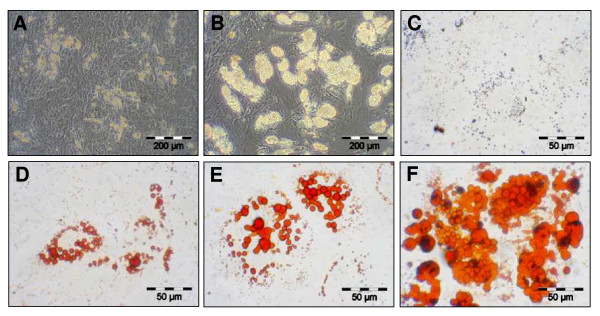
**Adipogenic differentiation potential of human MSC (A-F)**. First the adipogenically induced human MSC lipid droplet formation became visible at day 5 (A). The number and size was continuously increasing until day 15 (B). Control cultures, even at day 15, showed no lipid droplet accumulation (C). Oil red O staining of lipid-droplets clearly confirmed adipogenesis at day 5 (D), 15 (E) and 20 (F).

**Figure 4:**
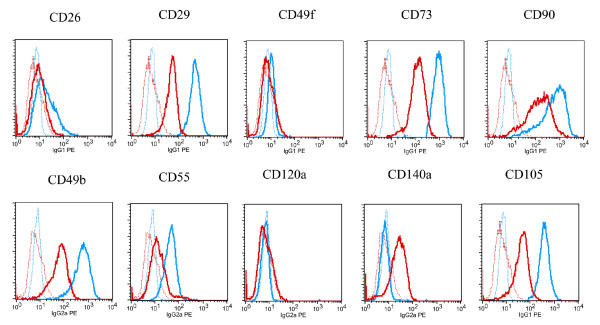
**Control of adipocyte differentiation by FACS studies**. The adipocytic differentiation was also monitored using flow cytometry with different markers. After growing 14 days some of the cells were harvested and marked with the monoclonal antibodies CD26, CD29, CD49b, CD55, CD73, CD90, CD140a (all Becton Dickinson, San Jose, CA), CD49f, CD120a (both Serotec, Raleigh, NC) and CD105 (Caltag, Buckingham, UK). As a negative control, cells were incubated with the corresponding irrelevant mouse IgG1, IgG2a, IgG2b or IgGM mAbs - therefore, cells became visible in the background. But only if a gene expression is changing in relation to the background after 14 days, can the result be positive. In Figure 4 the cells were colored early in the beginning with a blue marker and after 14 days with a red coloration. Only mAb CD140a clearly showed an increased gene expression after 14 days.

Expanded MSC were routinely tested for their multilineage potential by inducing these cells towards osteogenic, chondrogenic and adipogenic differentiation. Osteogenic differentiation could be demonstrated in the osteogenesis assay by staining for alkaline phosphatase (AP) activity as well as by intense von Kossa staining of a calcified bone matrix. On day 21, induced cultures showed clear signs of enhanced AP activity (Figure 2-A) and also an increased secretion of mineralized bone matrix (Figure 2-C) compared to controls (Figures 2-B, D). High-density micromass culture for chondrogenesis resulted in the formation of cartilaginous extracellular matrix as demonstrated by alcian blue staining of cartilage glycosaminoglycans (Figure 2-E) and antibody staining of cartilage-specific collagen type II (Figure 2-G). On day 21, non-induced control cultures did not show chondrogenic characteristics (Figures 2-F, H). Finally, before performing microarrays of adipogenic induced MSC, these cells were tested for their adipogenic potential. Lipid droplets were visible first after 3 to 5 days of growth in inducing media (Figure 3-A). Number and size of the droplets increased over the cultivation time of 21 days (Figure 3-B). Control cultures showed no lipid droplet formation (Figure 3-C). As demonstrated by oil red O staining of lipid droplets, the induced cultures showed an increased accumulation of cells with lipid droplets and the number and size of droplets per cell had strikingly increased (Figures 3-D, E, F). The adipocytic differentiation was monitored using flow cytometry with different markers (Figure 4) - see also Methods. After 14 days, expression of the mesenchymal markers CD26, CD29, CD49b, CD55, CD73, CD90, CD49f, CD120a and CD105 did not change or was reduced comparing to the beginning and only expression of CD140a had increased [17].

### Differential gene expression profiling of human MSC during adipogenesis

To investigate the adipogenic differentiation program of human MSC, we performed differential gene expression profiling using the genome-wide Affymetrix GeneChip technology. Three adult MSC donors and three native fat cell donors were used in this study and up-loaded to the online chip comparison database https://www.sipagene.de/sipagene. We compared the gene expression profiles of 3 individual group samples of adipogenically induced P3 human MSC grown in adipogenic media (passage 3) of days 1, 3, 7 and 17 with the undifferentiated MSC samples of day 0 (36 pairwise chip comparisons). Additionally, we compared the gene expression profiles of 3 samples of human fat tissues with the gene expression profiles of the corresponding MSC on day 0 (9 pairwise chip comparisons). Comparative testing of small groups is difficult. Using several statistical measures of the database at the same time, we tried to overcome this limitation - for example the percentage of increased or decreased change calls in all 3 × 3 = 9 pairwise comparisons between the individual chips of each group, the mean of the SLR and the corresponding Fold Change, p-values of 4 different t-tests and information derived from Chip signal calculations including mean -, median -, std - values (see also Methods) as well as present calls. Candidate genes were selected based on significance in t-test and increase or decrease - at least in one group comparison, including present call in the group of increased expression. Finally, five group comparisons (five experimental versus one baseline group) were performed.

Subsequently, as part of a two-step approach (see Methods), we identified genes with either consistently increased or consistently decreased expression in each of the five group comparisons and therefore are of interest in the context of adipogenesis. Among the 54,675 probe-sets of the genome-wide GeneChips we found 184 genes to be differentially expressed in a highly significant way (p < 0.00001, see Additional files 1 and 2). From this group 85 genes had increased expression values (Additional file 1) and 99 genes showed decreased expression values (Additional file 2), therefore both files represent candidate genes of interest in adipogenic development.

To check for correct annotation of the target sequences of the identified **probe sets **shown in Additional file 1 (increased values) and Additional file 2 (decreased values) we compared the sequences to entries of the RefSeq release 46 using BLAST http://blast.ncbi.nlm.nih.gov/Blast.cgi and Aligner (CodonCode Corporation). Most target sequences mapped with 100% match to the 3' end of the corresponding RefSeq entries for mRNAs. For some probe sets the mapping indicated that polyadenylation may occur upstream of the target sequences, may include intron sequences or may even locate outside of sequences referenced to known genes and transcripts - see Additional file 3 - part A (increased genes)/part B (decreased genes).

**Figure 5:**
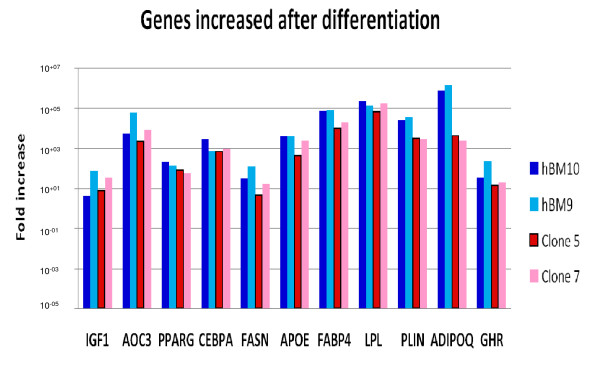
**Quantitative RT- PCR-analysis**. RNA from primary layers and clones was extracted before differentiation induction (day 0) and 14 days after addition of the adipogenic media. Here we tested the discussed adipogenic marker genes IGF 1, AOC3, PPARG, CEBPA, FASN, APOPE, FABP4, LPL, PLIN, ADIPOQ and GHR for up-regulated expression using quantitative RT-PCR-technology. For gene names see Additional file 1.

Finally, we confirmed by quantitative RT- PCR that the gene expression of known adipogenic marker genes were increased in adipocytes, generated not only from primary layers, but also from strictly individualized clones (Figure 5). The clonogenic cells generating the clones were bonafide MSC since they were also able, under appropriate conditions, to differentiate into osteoblasts and chondrocytes [2].

### Cluster analysis of adipogenic gene expression profile

**Figure 6:**
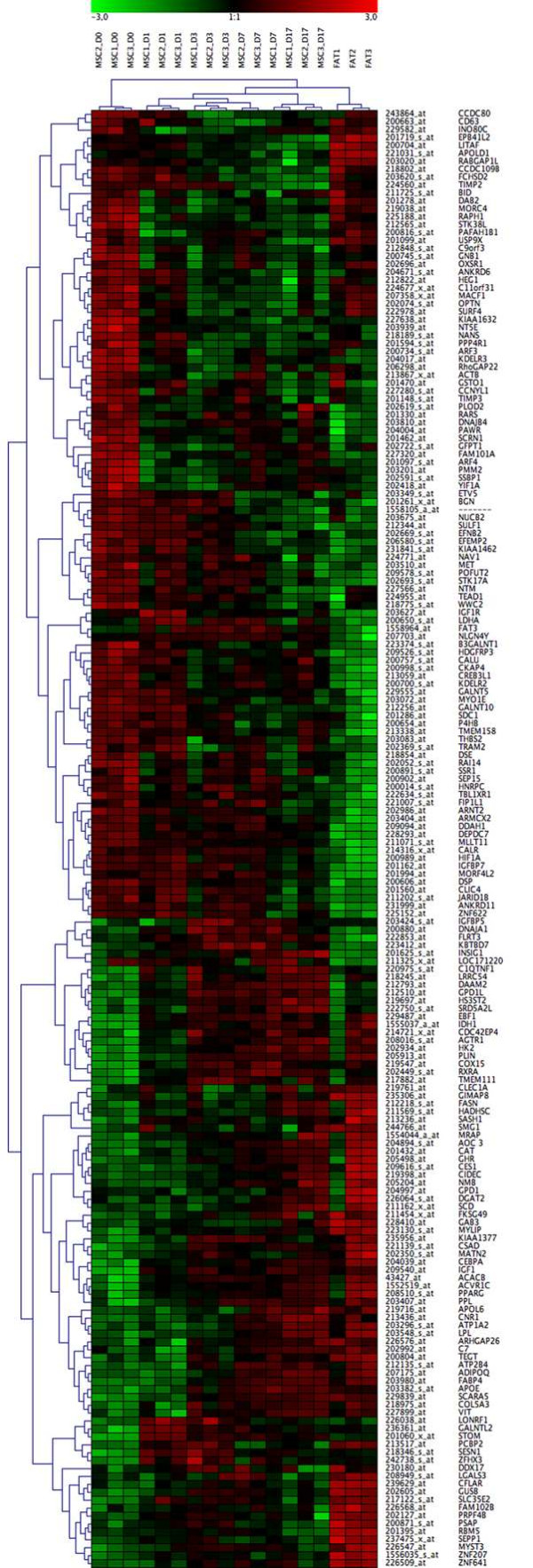
**Hierarchical cluster analysis of adipogenic gene expression profile**. Hierarchical cluster analysis of the 184 candidate genes (85 with increased expression, 99 with decreased expression) selected as relevant by SiPaGene. Clustering of genes and experiments resulted in two main groups, namely undifferentiated MSC at day 0 and all other experiments. The latter main group was subdivided in the native fat tissue group and the group of adipogenic induced MSC at day 1, 3, 7 and 17. The MSC group was further divided in two groups: in the first, we found all MSC cultures induced for 1 and 3 days (early adipogenesis) and 2 out of 3 MSC cultures induced for 7 days. In the second subgroup, we observed all MSC cultures stimulated for 17 days (later adipogenesis) and the third day 7 culture (see also the dendrogram). Colours represent relative levels of gene expression: bright red indicates the highest level of expression, and bright green indicates the lowest level of expression. On the right site gene names are included.

Hierarchical cluster analysis of genes was performed using the clustering tool GENESIS. As shown in Figure 6, for all 184 differentially expressed genes, undifferentiated MSC (day 0) clustered in one main group while MSCs cultured under adipogenic conditions and the native fat cell tissues clustered into another. This latter group further sub-divided into two groups, one consisting of MSC induced to differentiate into adipocytes, and the other consisting of native fat tissue samples. The group of adipogenic-induced MSC was also further sub-divided into two groups, representing either MSC induced for 1, 3, 7 or 17 days. Based on these 184 candidate genes, it was possible to detect striking differences in gene expression characteristic of adipogenic differentiation. Gene clusters were found to discriminate undifferentiated stem cells (day 0 of MSC) from early committed cells (MSC cultured cells in adipogenic media for 1-3 days), cells in an advanced stage of differentiation/maturation (MSC cultured in an adipogenic medium for 17 days), and fully differentiated adipocytes (found in native fat tissues). Genes characteristic of the different steps of differentiation are presented in detail in Additional files 1 and 2. Since the variability of the experimental groups was not very high, further statistical analysis was performed only using mean values of the genes with selectively increased and decreased expression levels.

### K-Means clustering and database - assisted biochemical pathway analysis

**Figure 7:**
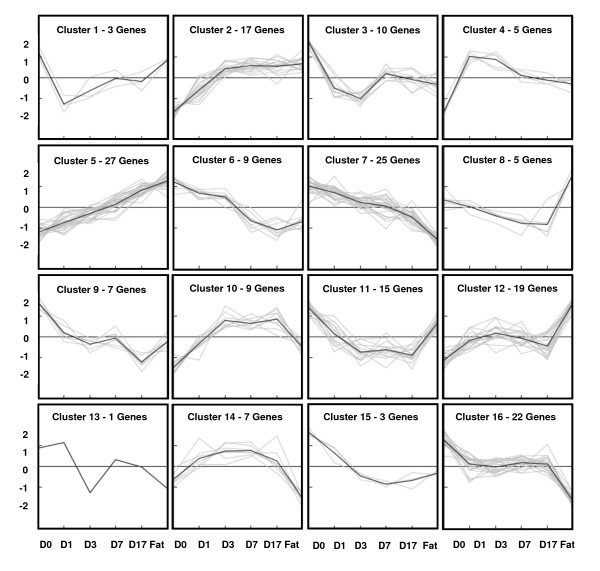
**K-Means cluster analysis of adipogenic gene expression profile**. 184 candidate genes possibly associated with adipogenesis were clustered into 16 different groups (C: Cluster) depending on the individual expression levels (K-means clustering method). The intensity is shown by numbers on the left border, the expression levels are illustrated by means of the cluster images.

**Figure 8:**
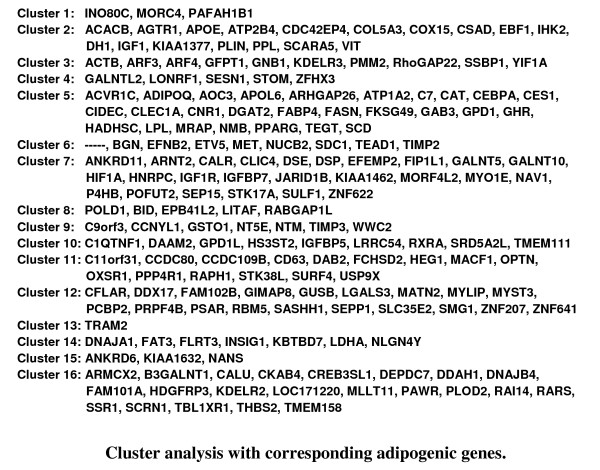
**Cluster analysis with corresponding adipogenic genes**. The number and names of the corresponding Cluster genes are listed in Figure 8. It became possible to observe the expression profiles of different cluster genes (known or unknown for adipogenesis) and to find further early and late stage adipogenesis marker genes with similar qualities. Detailed mean signal values of human MSC at day 0, 1, 3, 7, 17 and of native fat tissue are also given in Additional files 1 and 2.

For the 184 genes that were selected (Additional files 1 and 2), two different follow-up analysis strategies were used: K-means clustering and biochemical pathway analysis. Applying K-means, 184 genes were clustered according to a similar course of expression. The number of clusters was estimated by Figure-of-Merit analysis [18]. Sixteen different clusters could be determined, each of them composed of 1-27 different genes. Applying this strategy, we directly compared all signal values of various kinetic expression profiles. Here it was possible to analyze the expression status of human MSC during adipogenesis and in comparison with native fat tissue. Moreover, groups of genes with a similar expression profile could be identified (Figure 7, Figure 8).

It was also feasible to observe the expression profiles of different cluster genes (known or unknown for involvement in adipogenesis) and to find further early and late stage adipogenesis marker genes with similar qualities. For example, cluster 5 included the early stage adipogenesis genes *PPARG *as well as *ADIPOQ*, while in cluster 2 the very early stage adipogenesis gene *PLIN*, but also *IGF 1 *was found. The difference between both clusters was demonstrated by the classic kinetic shape of their expression profiles. For the biochemical pathway analysis, 85 increased expression adipogenic candidate genes were loaded into the databases DAVID [19] and KEGG [20], and then further divided into sub-groups (e.g. DAVID) or just analyzed (e.g. KEGG and iHOP [21]) depending on their known function, such as PPARG signalling or fatty acid biosynthesis (Table 1). A few strongly expressed candidate genes could not be integrated into our group system according to their already known biochemical function. Here of course it is interesting to discuss their possible role during adipogenesis. Sometimes, candidate genes with decreased expression values do interact with known biochemical pathways, but quite often we do not find a logical explanation in terms of adipogenesis.

**Table 1:** Adipogenic candidate genes and their involvement in biochemical pathways.

Pathway	Candidate genes
**PPARG signalling pathway**	Up-regulated:*RXRA, C/EBPA*, *CES1*, *PPARG, GPD1*
**Adipocyte differentiation pathway**	Up-regulated:*FABP4*, *PLIN*
**Adipocytokine signalling pathway**	Up-regulated:*LPL, ADIPOQ, APOE, LGALS3, COL5A3*, *APOL6, CLEC1A, FLRT3*
**Lipogenesis pathway**	Up-regulated:*HADHSC, SCD, FASN, DGAT2*, *SRD5A2L, ACACB*
**Insulin-like signalling pathway**	Up-regulated:*IGFBP5, INSIG1, IGF1R, GHR*, *IGF1* Down-regulated:,*IGFBP7*
**Apoptosis(factors related to, or no pathway)**	Up-regulated:*ACVR1C, AOC3, C1QTNF1, C7, CFLAR, CIDEC, DDX17, LASP8, PI-3-kinase, RBM5, SMG1* Down-regulated:*BID, HNRPC, MORF4L2, PAWR, SULF1*

The 184 selected genes were analyzed in different online databases (DAVID, KEGG and iHOP), for example in gene studies and the gene products were subsequently in biochemical pathway studies performed. Genes, up-regulated during adipogenesis were of most interest. This Table 1 shows pathways and corresponding genes. For gene names and mean signal values see the Additional files 1 and 2. Charts that illustrate expression values as a function of time are presented in Figure 7 and Figure 8.

Therefore our main focus was on up-regulated genes: only some of the decreased genes will be discussed here and are shown in Table 1 or in Figure 7 and 8. Most likely, these down-regulated genes inhibit or repress the outcome of biochemical interactions during the process of adipogenesis. In the following, we will highlight some of the most interesting pathways involved in adipogenesis.

### The PPAR-Gamma signalling pathway

We found at least three essential transcription factors of the PPARG signalling pathway to be up-regulated: the *CCAAT/enhancer binding protein α *(*C/EBPA*), the *peroxisome proliferator activated receptor γ *(*PPARG*) and the *retinoid x receptor *α (*RXRA*). We already identified direct targets such as *carboxylesterase 1 *(*CES1*) and *Gycerol-3-phosphate dehydrogenase 1-like *(*GPD1*) to be up-regulated (see Table 1).

### Additional genes implicated in adipogenesis

We also found up-regulation of at least three subsequent adipogenic target pathways: (1; Table 1) here just called genes implicated in early adipogenesis, such as the **adipocyte differentiation pathway **with the two very early fatty acid binding proteins *perilipin *(*PLIN*) and *fatty acid-binding protein 4 *(*FABP4*), as well as (2; Table 1) *adiponectin *(*ADIPOQ*), a hormone which is thought to be active further downstream in the **adipocytokine signalling pathway **and, subsequently, in the differentiation process. Furthermore, we found up-regulated expression of genes involved in fatty acid transport such as fatty acid transport with the hormone and receptor protein *apolipoprotein E *(*APOE*) as well as *apolipoprotein L, 6 *(*APOL6*), *collagen, type V, -alpha 3 *(*COL5A3*), *fibronectin leucine rich transmembrane protein 3 *(*FLRT3*), *lectin galactoside-binding, soluble, 3 *(*LGALS3*) and *c-type lectin domain family 1, member A *(*CLEC1A*) as well as the classic transport and reducing enzyme *lipoproteinlipase *(*LPL*). We also detected up-regulated expression of factors involved in triggering **lipogenesis **(3; Table 1), such as *stearoyl-CoA-desaturase *(*SCD*), *acetyl-coenzyme A carboxylase beta *(*ACACB*), *diacylglycerol-O-acyltransferase homolog 2 *(*DGAT2*), *fatty acid synthase *(*FASN*), *L-3-hydroxyacyl-coenzyme A-dehydrogenase short chain *(*HADHSC*), and *steroid 5 alphareductase 2-like *(*SRD5A2L*).

### The Insulin-like signalling pathway

Furthermore, genes encoding signalling cascades that are involved in carbohydrate metabolisms as well as in cell growth and cellular interactions were also found to be up-regulated. Accordingly, during the observed time period a strongly up-regulated expression was found for the human MSC *growth hormone receptor *(*GHR*) as well as for some transcription factors of the **insulin-like signalling pathway **(4, Table 1) such as the *insulin-like growth factor 1-somatomedin C *(*IGF1*), *insulin-like growth factor 1 receptor *(*IGF1R*), *insulin induced gene 1 *(*INSIG1*), *insulin-like growth factor binding protein 5 *(*IGFBP5*), and the *insulin-like growth factor binding protein 7 *(*IGFBP7*).

### The Apoptosis pathway

We detected a whole set of apoptotic factors which were up- or down-regulated during adipogenesis. **Apoptosis factors **or modulators with increased expression during adipogenesis were the *activin A receptor type IC *(*ACVR1C*), *complement component 7 *(*C7*), *PI-3-kinase-related kinase SMG-1 *(*SMG1*), *CASP8 *and *FADD-like apoptosis regulator *(*CFLAR*), *amine oxidase copper containing 3 *(*vascular adhesion protein 1*) (*AOC3*), *cell death-inducing DFFA-like effector c *(*CIDEC*), *DEAD (Asp-Glu-Ala-Asp) box polypeptide 17 *(*DDX17*), *C1q and tumor necrosis factor related protein *(*C1QTNF1*) and *RNA binding motif protein 5 *(*RBM5*). We also found at least five apoptosis factors with decreased gene expression including *PRKC apoptosis WT1 regulator *(*PAWR*), *BH3 interacting domain death agonist *(*BID*), *mortality factor 4 like 2 *(*MORF4L2*), *heterogeneous nuclear ribonucleoprotein C *(*C1/C2*) (*HNRPC*) and *sulfatase *1 (*SULF1*).

### Other molecules that have not yet been described in the context of adipogenesis

Finally, new candidate genes coding for proteins and possible regulatory factors, which may be related to the adipogenesis of human MSC, have been identified. In particular, strong up-regulation of gene expression was found for the human receptor protein *scavenger receptor class A member 5*-*putative *(*SCARA5*) with a putative transmembrane domain and a collagen-like domain, and the endoplasmic reticulum protein *melanocortin 2 receptor accessory protein *(*MRAP*), and as well as some zinc finger receptors (ATPases or GTPases) such as the proteins *IMAP family member 8 *(*GIMAP8*).

## Discussion

In this work we have isolated from bone marrow aspirates human mesenchymal stem cells characterized by their surface marker profile [1,17] and their osteogenic, chondrogenic and adipogenic potential [5-7,22,23] to study the expression profile of genes involved during maturation and adipogenic differentiation. These cells showed all the important characteristics of human MSC and were further used for gene expression profiling analysis during adipogenic development. In studies of human mesenchymal tissue development, the analysis of expression of specific genes, carried out over a distinct time period, is relevant to understanding the maturation process. Since the applied cocktail of adipogenic inducers, dexamethasone, 3-isobutyl-1-methylxanthine, indomethacin and insulin, was sufficient to promote differentiation of human MSC to adipocytes, these MSC were further used for gene expression profiling during adipogenesis. Our data helped identifying gene sets characterizing the sequential differentiation steps from stem cells to fully differentiated adipocytes. In our study, we observed differentially expressed genes coding for molecules that have not yet been described in the context of adipogenesis. For example the expression of human receptor gene *SCARA5 *and the endoplasmic reticulum protein gene *MRAP *were highly up-regulated, suggesting new candidate genes effective in adipocytic differentiation. Also we have confirmed most of the already described marker genes involved in adipogenesis [4].

The PPARG signalling pathway has been intensively studied over several years and scenario through which various adipocyte transcription factors interact with adipogenic target genes have already been proposed [24]. PPARG is a hormone receptor, which has already been demonstrated to be the master regulator of adipogenesis [25,26]. The dependent downstream-regulation cascade can be controlled by PPARG itself or by PPARG in combination with the controlling factor C/EBPA (also up-regulated in our study).

It is very likely that C/EBPA and PPARG direct the final phase of adipogenesis by activating expression of adipocyte-specific genes, such as fatty acid synthetase, fatty acid binding protein, leptin and adiponectin. However, the mainstream interaction between PPARG as well as the RXRA receptor, another gene product was found to be up-regulated during adipogenesis in our study via ligand-binding, results in a conformational change, namely in the formation of a special heterodimeric protein structure. The remaining functional domain within the complex binds to various peroxisome proliferator response elements (PPREs) and therefore activates transcription of adipogenic target genes via phosphorylation events, or, alternatively, causes induction of co-activator and co-repressor complexes [27,28]. It has been claimed that about 200 proteins can be controlled by PPARG, probably via the PPARG-RXRA signalling pathway [29]. Therefore, it was not surprising that various biochemical subgroups belonging to carbohydrate metabolism, citrate cycle and energy transfer or ion transport could be reformed on the basis of the identified adipogenic candidate genes. Interestingly, we found the PPARG target CES1, an enzyme located in the endoplasmic reticulum and responsible for cellular detoxification [30] to be highly up-regulated. Another PPARG target example is presented by the addition of a second GPD1 enzyme type. The PPARG-RXRA signalling expression pattern leads to several additional downstream adipogenic target genes and corresponding pathways, which are either up- or down-regulated [31]. Of course, we also found other up-regulated PPARG targets, such as the transcripts coding for fatty acid-binding FABP4 [32] and PLIN, which binds to fatty acids in such a way that lipid droplets are formed and fatty acids are protected against degrading enzymes [33]. Furthermore, the mobilization of the stored triglyceride is thought to be controlled by interactions among intracellular lipases during hormone-mediated lipolysis and other proteins that coat lipid storage droplets [34]. In our study, the mean value of PLIN was greatly increased after 17 days as was that of the hormone ADIPOQ, which is also a known downstream target of the PPARG-RXRA-regulator. Recently, PPARG has received attention as a possible pharmacological target for the thiazolidinedione class of anti-diabetic drugs as it is essential for the final phase of adipocyte differentiation and the pro-adipogenic effects of thiazolidinediones have spurred interest in identifying therapeutic compounds that retain anti-diabetic activity without promoting adipogenesis. Inhibitors of PPARG activity that reduces adipogenesis and thus serve as the basis for the development of effective anti-obesity drugs [31] have already been identified.

Further highly expressed genes (all named by the gene name) were grouped together according to their known biological function and pathway. The lipogenesis group includes *fatty acid synthase *(*FAS*), which synthesizes fatty acids (responsible for lipid accumulation). This group also includes the enzymes *ACACB, SRD5A2L*, *HADHSC*, and *DGAT2*. *ACACB *is a biotin-containing enzyme, which catalyzes the carboxylation of acetyl-CoA to malonyl-CoA, the rate-limiting step in the fatty acid synthesis. But ACACB is also a complex multifunctional enzyme system, which controls fatty acid oxidation through the ability of malonyl-CoA to inhibit carnitine-palmitoyl-CoA transferase I.

As expected we found the adipokine and adiponectin metabolism (ADIPOQ) to be up-regulated. *ADIPOQ *encodes a central cytokine, which is released by adipocytes. It is involved in the control of fat metabolism and insulin sensitivity, with direct anti-diabetic, anti-atherogenic and anti-inflammatory activities. In the human body, adiponectin stimulates the phosphorylation of the AMP activated protein kinase (AMPK) in liver and the skeletal muscle, antagonizes TNF-α by negatively regulating its expression in various tissues such as liver and macrophages, and also by counteracting its effects in adipocytes by enhancing glucose utilization and fatty-acid production [35]. The inhibitory potential of TNF-α is mediated using the endothelial NF-kappa-B signalling cAMP-dependent pathway [36,37]. ADIPOQ also plays a role in cell growth, angiogenesis and tissue remodelling by binding and sequestering various growth factors. It is very likely that adiponectin interacts with one of the collagens or is part of the extracellular matrix system [38]. At least one of the known collagen receptor genes, *COL5A3*, was slightly up-regulated in our study. We also detected the up-regulation of typical cell-cell signalling factor genes with adhesive/cytokine like function, such as *CLEC1A *and *LGALS3*. It has been shown that LGALS3 can protect mitochondrial membrane integrity and models suggested that it acts as an anti-apoptotic factor, because it prevents cytochrome C release, thereby blocking the effector stage of apoptosis [39]. It was discussed in literature that APOL6 can contribute to the formation of ion channels through intracellular membranes and is therefore involved in mechanisms triggering programmed cell death [40]. In any case, the regulator hormone ADIPOQ consists of a collagen domain by itself and can pass through different cellular membranes without any alteration of the domain. ACACB is also part of the adipocytokine signalling pathway and seems to play a central enzyme role in rats as well as in man and alterations of the expression pattern can possibly be responsible for obesity, diabetes [41], and other metabolic pathway activities.

In addition, cytokines such as tumor necrosis factor alpha (TNF-α) and other transforming growth factors interfere with adipocyte differentiation. As expected, not all typical examples for adipocytokines (e.g. leptins, TNF-α, Interleukin-6 (IL6) or resistin) are expressed in all types of tissues identically [42] - and some adipocytokines were not found in this study. Nevertheless, we detected members that are involved in the adipocytokine-signalling pathway such as *FLRT3*, of which the protein product might also function as a receptor signalling protein as well as a cell adhesion protein [43].

During the adipogenic maturation process, media containing insulin may have resulted in the development of insulin-independent mechanisms [44]. This phenomenon probably was the reason why the insulin-independent cellular membrane receptor, which is called *glucose transporter-member 1 *(*GLUT1*), was missing in our candidate gene list. Instead we found up-regulation of the lipoprotein gene *stomatin *(*STOM*). Over-expression of this lipoprotein often results in depressing the basal rate of glucose transport by decreasing the intrinsic activity of GLUT1 [45]. However, we also detected insulin-dependent genes, such as *INSIG1*, which have been suggested as a target of PPARG [4].

During adipogenic differentiation, we found that the expression of *IGFBP7 *was down-regulated. The insulin-like growth factor binding family is thought to modify most IGF1 actions [46] and is very important for human growth, while insulin by itself can bind to the IGF1 receptor to activate the phosphatidyl-inositol-3-phosphat kinase (PI3K)/Akt (proteinkinase B)-signal mechanism of the cellular structure [47]. However, a few members of IGFBP family, such as IGFBP7, do have other biochemical functions. For example IGFBP7 also binds directly to insulin and in an artificial cell system it was recently demonstrated that glioma cell growth can be mediated by expressing *IGFBP7 *[48]. This observation fits very well with our second finding, and therefore we assume again that IGFBP7 has a negative effect on adipocyte differentiation. IGFBP7 can modulate the stimulatory effect of vascular endothelial growth factors (VEGF) on angiogenesis by interfering with VEGF expression [49]. On the other hand, IGFBP5 is thought to be associated with proteins of the extracellular matrix (ECM) like fibronectin depending on bound molecules, which might interfere with IGF1 and further growth activities [46,49].

Strikingly, we detected increased expression of members of the angiotensin II modulator family, such as the *angiotensin II type 1 receptor *(*AGTR1A*) gene, coding for a receptor whose action is mediated by association with G proteins, which activate the phosphatidylinositol-calcium second messenger system. The human rennin-angiotensin system can be activated by AGTR1A, while angiotensin II is the main regulator or effector of the cellular proliferation and survival [50]. Recently, many publications demonstrated that tissue specificity is achieved by modulation of the angiotensin receptor proteins together with effector proteins [51], which would fit well with our data and implies that *AGTR1A *is involved in the adipocyte development. We also found up-regulation of a further G protein-coupled receptor gene, *the cannabinoid receptor 1 *(*brain*), called *CNR1 *[52]. The CNR1 receptor is the most abundant G protein-coupled receptor expressed in the brain. However, CNR1 has also been detected in adipocytes, which is consistent with our data, suggesting that CNR1 is directly or indirectly effective on adipogenesis.

The regulatory factor *lipopolysaccharide-induced TNF-α factor *(*LITAF*), which was down-regulated during adipogenesis until day 17 and expressed at much lower levels when compared with native fat cell tissue, might play an important modifying role in TNF-α gene expression either through induction of lipopolysaccharides (LPS) or via using the apoptotic TP53/p53 pathway. Moreover, some fatty acids act as signalling molecules regulating the differentiation into adipocytes or cell death.

We also found up- or down-regulation of a number of transcripts coding for factors implicated in apoptosis such as *LITAF*, *PRKC, PAWR*, *BID, MORF4L2*, and *HNRPC*. It has been shown very recently that LITAF is a transcription factor, which plays an important role in regulating the expression of TNF-α and various other inflammatory cytokines in response to LPS stimulation, negatively affecting adipocytic regulation [53], but this observation still has to be confirmed. With our comparison approach we demonstrated that several other apoptotic factors or mediators have been increased or decreased during adipogenesis of human MSC. The *PAWR *(*PRKC, apoptosis, WT1, regulator*) expression was down-regulated as well. It has regulatory control functions via down-regulating the *B-cell lymphoma 2 *(*BCL-2*) gene expression. The *BH3 interacting domain death agonist *(*BID*) was also down-regulated and is responsible for the release of mitochondrial cytochrome c. Two further down-regulated transcription factor candidates, which do both belong to a part of the apoptosis pathway, are the transcription factor genes *mortality factor 4 like 2 *(*MORF4L2*) and the *heterogeneous nuclear ribonucleoprotein C *(*C1/C2*) (*HNRPC)*.

We detected the up-regulation of genes, whose encoding factors yet are unrelated to adipogenesis, such as *SCARA5 *and *MRAP*. It is already known that SCARA5 is a ferritin receptor mediating non-transferrin iron delivery, which is essential for cell growth [54], and that MRAP might control cellular trafficking with the help of other transmembrane proteins [55]. Our data suggest their positive effect on adipogenesis, which should be further investigated in gain and loss of function studies. We also observed up-regulation of central genes coding for enzymes of the glycolytic pathway such as hexokinase 2 (HK2), which is integrated in mitochondrial membranes instead of working in the cytosol [56], as well as many stress proteins such as the peroxisome control enzyme catalyze (CAT).

As a follow-up strategy it will be important to identify regulatory networks related to specific transcription factors. Search tools like rVISTA [57] suggest further regulatory proteins or transcription factors based only on potential binding sites upstream of the genomic sequence of the named genes and calculate the probability that individual binding sites are identified with a significantly increased frequency. For example, the list of our up-regulated candidates (Additional file 1) returned GATA3, HFH3, AP4, FOXO4, POU6F1, FOXO1, GRE, ISRE, LBP1, FOXP3 as potential candidates involved in the regulation of these genes. However, this approach can only generate hypotheses, which require further confirmation by other techniques like chromatin immunoprecipitation followed by array hybridisation. It also will be interesting to check, if some of the detected adipogenic genes of the defined clusters or from one biochemical group (see Table 1) perform a familiar pattern of transcriptional regulation or to find *de novo *binding strategies on the promotor site of the genes in further experiments.

## Conclusions

Adipogenesis is the developmental process by which multipotent MSC differentiate into mature adipocytes. We confirmed observations by others that several molecular factors and biochemical pathways are involved in this complex process of adipogenesis. However and to go beyond the state-of-art, we analyzed the entire gene expression profiles of human MSC during adipogenesis over a defined time scale and compared the findings with the expression profile of native fat cell tissue as a reference. In this regard our emphasis was on differentially expressed genes coding for key molecules and transcription factors of fat cell development and on the involvement of such factors in biochemical signalling pathways. A battery of known (*PPARG*, *C/EBPA *and *RTXA*) and new (*SCARA5 *and *MRAP*) adipogenic marker genes was found, and some of the newly identified marker genes were linked to specific biochemical signalling pathways.

Using the online chip database SiPaGene it was possible to establish a comprehensive kinetic gene expression system and a network of adipogenesis genes, which otherwise could only be found using multiple special search approaches.

## Methods

### Isolation and expansion of human MSC

Human mesenchymal stem cells were isolated from iliac crest aspirates obtained from informed and consenting patients undergoing orthopedic surgery (Trousseau Hospital, Tours, France) or diagnostic examinations (examined to exclude hematopoietic neoplasmas and histologically diagnosed as normal; Charité-Universitätsmedizin, Berlin, Germany), following a procedure approved by the local ethical committees. As previously described [17], nucleated cells were counted and seeded at a density of 5 × 10^4 ^per cm^2 ^culture surface in α-MEM (Invitrogen) supplemented with 10% (v/v) screened fetal bovine serum (FBS), 1 ng/ml basic-fibroblast growth factor (basic-FGF; AbCys, Paris, France), 20 μmol/l L-glutamine (Invitrogen) and 100 units/ml penicillin G (Invitrogen). Cells were incubated under standard culture conditions. On day 3, non-adherent cells were removed by changing the medium; thereafter, the medium was changed twice a week. After reaching confluence, cells were trypsinized (0.25% v/v trypsin EDTA; Invitrogen), re-suspended in culture media and seeded at 1 × 10^3 ^cells per cm^2 ^(passage 1, P1). The isolation of MSC clones was performed as previously described [2].

### Isolation of native fat tissue

Fat tissue samples were harvested from three informed and consenting patients with osteoarthritis of the knee during joint replacement surgery from healthy areas of normal subcutaneous fat tissue. The donation of fat tissue was approved by the ethical committee of the Charité-Universitätsmedizin Berlin.

### Flow cytometry analysis

To verify the presentation of typical human MSC marker and a lack of remaining hematopoietic CD45^+ ^cells, flow cytometry analysis was routinely performed on culture amplified human MSC. Cells were collected from confluent layers at the end of P2 after incubation with 0.25% trypsin-EDTA for 5 minutes. Single-cell suspensions were washed with phosphate buffered saline (PBS)/0.5% bovine serum albumin (BSA) before staining. For direct staining, cells were centrifuged (250 g, 5 min) and re-suspended in cold PBS/0.5% BSA. 2.5 × 10^5 ^cells were incubated for 15 minutes on ice in the dark in cold PBS/0.5% BSA with titrated concentrations of R-phycoerythrin (PE) conjugated monoclonal mouse anti-human CD34, CD73, CD166 antibodies or monoclonal fluorescein isothiocyanate (FITC) conjugated mouse anti-human CD45, CD90, CD105 antibodies. All antibodies except CD105 were purchased from BD-Pharmingen (Heidelberg, Germany). Monoclonal CD105 was purchased from Acris Antibodies (Hiddenhausen, Germany). Cells were then washed twice by centrifugation (250 g, 5 min) and re-suspended with cold PBS/0.5% BSA, before proceeding with flow cytometry analysis. Dead cells and debris were stained with propidium iodide (100 μg/ml; Sigma-Aldrich, Taufkirchen, Germany) and excluded from measurements. Acquisitions were performed on a FACS Calibur flow cytometer (Becton Dickinson) and data was analyzed using CellQuest 3.3 software (Becton Dickinson) [17].

### Verification of MSC multilineage potential

The multilineage differentiation potential of human MSC (n = 3, P3) was analyzed by applying standard protocols used by Pittenger et al. [1]. For adipogenesis 10,000 MSC/cm^2 ^were seeded. Five days after reaching confluence, MSC were treated for three days with induction media consisting of DMEM (4.5 g/l glucose; Biochrom, Berlin, Germany) supplemented with 10% FBS, 1 μM dexamethasone (Sigma-Aldrich), 0.2 mM indomethacin (Sigma-Aldrich), 10 μg/ml insulin (Actraphane, Novo Nordisk, Bagsvaerd, Denmark), 0.5 mM 3-isobutyl-1-methylxanthine (Sigma-Aldrich), and then for two days with maintenance media consisting of DMEM (4.5 g/l glucose), FBS and 10 μg/ml insulin.

This cycle of three days of induction and 2 days of maintenance was repeated three times. Control cells received only maintenance media. Osteogenesis was induced for up to 28 days in Iscoves's/DMEM/Ham's F12 medium (Biochrom) supplemented with 1% FBS, 100 nM dexamethasone, 10 mM β-glycerolphosphate (Sigma-Aldrich) and 0.05 mM L-ascorbic acid 2-phosphate (AsAP; Sigma-Aldrich). Control cells were cultured in media without the latter three substances. To form high-density micromass cultures for chondrogenic induction, 2 × 10^5 ^MSC were centrifuged and subsequently cultured for up to 28 days in a defined medium consisting of DMEM (4.5 g/l glucose), ITS+1 (Sigma-Aldrich), 100 nM dexamethasone, 0.17 mM AsAP, 1 mM sodium pyruvate (Sigma-Aldrich), 0.35 mM L-proline (Sigma-Aldrich) and 10 ng/ml transforming growth factor-β3 (TGFβ3; R&D Systems, Wiesbaden, Germany). Controls were cultured without TGFβ3.

### Histological methods and immunohistochemistry

To demonstrate adipogenic differentiation, oil red O (Sigma-Aldrich) staining of lipid droplets was performed. To verify osteogenesis, visualization of alkaline phosphatase activity with Sigma fast BCIP/NBT (Sigma-Aldrich) and von Kossa staining (Sigma-Aldrich) of a bone-specific mineralized matrix, was carried out. Chondrogenesis was shown by staining of cartilage proteoglycans with alcian blue 8GX (Roth, Karlsruhe, Germany) counterstained with nuclear fast red (Sigma-Aldrich), and by immunohistochemistry using the EnVision+System, Peroxidase rabbit kit AEC (Dako). Cryosections (6 μm) were incubated for 1 h with primary rabbit anti-human type II collagen antibodies (Acris). Subsequently, samples were treated according to the manufacturer's protocol and counterstained with hematoxylin (Merck, Darmstadt, Germany).

### RNA isolation and microarray analysis

To gain RNA for gene expression profiling, human MSC from 3 different donors signed hBM19, hBM10, hBM9 (n = 3 donors, P1) were adipogenic stimulated for up to 17 days as described above. At day 0 (unstimulated MSC), 1, 3, 7 and 17, total RNA was isolated applying protocols of the RNeasy Mini Kit (Qiagen, Hilden, Germany) including DNAse digestion. To isolate RNA from three native fat tissues, 1 mg tissue was added to 1 ml TriReagent (Sigma-Aldrich) and mechanically homogenized using an Ultra-Turrax (IKA, Staufen, Germany). Then the RNeasy Mini Kit was used for RNA isolation. In general, the RNA was checked for integrity and purity with the Agilent Bioanalyzer (Agilent Technologies, Böblingen, Germany) and NanoDrop spectrophotometer (NanoDrop products, Wilmington, DE, USA).

Total RNA from each individual donor and sample was used for genome-wide gene expression profiling. Hybridization on HG-U133 Plus 2.0 oligonucleotide microarrays (in total 18 GeneChips) was performed according to standards supplied by the manufacturer (Affymetrix, Santa Clara, CA, USA). In brief, cDNA was synthesized from 5 μg of total RNA and submitted to *in vitro *transcription (ENZO, New York, NY, USA) to generate biotin-labelled complementary RNA. 15 μg of the fragmented complementary RNA were hybridized to the GeneChips for 16 hours at 45°C. Arrays were washed and stained under standardized conditions and scanned on a Hewlett Packard Genearray Scanner. Affymetrix GCOS 1.4 software was used to control washing and scanning, to generate DAT, CEL and EXP files and to process the raw data for signal calculation and pairwise chip comparison [16].

### Quantitative RT- PCR - analysis

RNA from primary layers and clones was extracted before differentiation induction (day 0) and 14 days after addition of the adipogenic medium. The MSC were lysed directly in the culture dishes and total RNA was isolated using RNeasy Kit (Qiagen, Chatsworth, CL). TaqMan low density arrays (Applied Biosystems, Foster City, CA, http://www.appliedbiosystems.com) were used in a typical quantitative two-step RT-PCR process as previously described.

### Gaining an adipogenesis expression profile of human MSC

Initially, data obtained from all 18 microarray experiments were processed with Affymetrix GCOS 1.4 software. In pairwise chip comparison analyses each of the three native fat tissue samples and of the three individual adipogenic stimulated MSC cultures on day 1, 3, 7 and 17, were compared with each of the three not induced MSC cultures on day 0 (5 [native tissue, adipogenic induced MSC on day 1, 3, 7, 17] × 3 [donors] × 3 [not induced MSC on day 0] = 45 pairwise chip comparisons).

Subsequently, GCOS signal values, detection calls and detection p-values (absolute data) as well as the signal log ratios, change calls and change p-values (pairwise chip comparison data) were uploaded into the online database SiPaGene, where single group comparisons and gene retrieval were performed [16]. This database has been developed for the storage or exchange of Affymetrix GeneChip microarray data and the performance of differential gene expression experiments like comparing the imported Affymetrix microarrays in special groups with each other and starting different statistical analyses. Based on the algorithms of the Affymetrix software and the Affymetrix standard settings for integrated parameters like detection call in case of chips and change call in case of pairwise chip comparison, the speciality when performing the analysis with SiPaGene is to scale the Affymetrix signal values using a target value of 150 for all arrays.

Our Affymetrix probe set annotation data set is always adapted in parallel to the newest provided version (version 46) - there is also an on-going discussion whether oligonucleotide sequences should be remapped to the latest genome build in order to avoid mistakes like ambiguous probe matching.

In more detail, for each of the selected five group comparisons (always consisting of one baseline group with the three donors of a not induced MSC culture at day 0 and of three experimental donors of either native fat cells or adipogenesis induced MSC at day 1, 3, 7 and 17) new statistical values were generated. Corresponding to the up-loaded Affymetrix data (absolute or comparative data) for each group, we used the following information:

the absolute signal, the mean value, the median value, the standard deviation and the present detection call, and for each group comparison as well, we used the total signal log ratio (SLR) and the total fold change (FC), the change call increase, the change call decrease and the no change values were calculated. Furthermore, Welch t-test analyses on signals between two groups as well as on SLR values of pairwise comparisons of chips within the same group or between groups were performed as part of each group comparison.

### Selection of candidate genes

With our strategy to perform the five different single group comparisons, we targeted building up a special differential kinetic group comparison profiling during adipogenesis. In this way, the progress of adipogenesis could be examined, including native fat tissue as a reference tissue. But we used a two step-approach to identify genes whose expression was increased or decreased in each of the five group comparisons and which are therefore of interest in the context of adipogenic development of human MSC. We expected that the gene expression of our candidate genes would be significantly increased or decreased in at least one comparison and be present at each of the five group comparisons.

During the first step, adipogenesis selection parameters were utilized to select from all 54,675 probe-sets of one comparison analysis, whereas during the second step, the selected increased or decreased candidate genes were analyzed in greater detail. Genes were selected as relevant only when fulfilling the specific standards. SiPaGene presents default query parameters to select increased expression data sets or decreased expression data sets. It also provides the usage of distinct query parameters for single group comparisons, for example to determine the minimum false discovery rate. In this study, we modified the queries for the default parameters of increased and decreased expression and compared as a very last step the findings with the results in accordance with default parameters of minimum false discovery (Bonferroni correction).

Concretely, we mainly analyzed the combination of the different statistical values (e.g.: mean value, median value) between the experimental group and the baseline group and also took into account parallel further combinational values like the increased or decreased value. To find genes whose expression was increased during adipogenesis, we used SiPaGene default parameters but additionally defined FC >= 2. Criteria for decreased expression were also the already defined default parameters with the additional criteria FC <= -2. In addition, the increased/decreased values had to be at least greater than 50% and the p-values of different Welch t-test analysis at least <= 0.00001. All candidate genes were verified by repeating the data analysis, but using different selection parameters or just default parameters of SiPaGene. Finally, the selected candidate genes were compared again with each other to remove double genes or probe-sets and to eliminate false positives via the Bonferroni correction.

### Further characterization of candidate genes

Genes meeting all the criteria were further analyzed by hierarchical clustering using GENESIS 1.7.2 software [18]. To find if our genes or gene products are important for adipogenesis, we loaded the list of relevant genes into the online databases DAVID [19] and KEGG [20], a resource linking genomes to life. Important information we have also collected with the environment and the literature research database iHOP [21].

In principle two main strategies were applied to detect information about candidate genes with increased or decreased expression. One possibility continued with functional clustering or K-means value analysis (kinetic comparison values) of gene clusters, while the second approach started with biochemical pathway analysis.

Quite often we searched for unknown genes or transcription factors, which showed a similar expression pattern to factors belonging to an already described or biochemical important pathway related to adipogenesis. Using functional clustering it often becomes easy to find unknown clusters of up- or down-regulated genes during a large scale selection or better genes or groups of genes, which lead to an identical biochemical pathway. Although we had already done a pre-selection with SiPaGene, we first used the cluster building strategy to confirm our results and afterwards tried to find more significant subgroups. Therefore all candidate genes detected using the cluster building strategy were further analyzed with help of public biochemical databases such as DAVID and KEGG or via literature databases like iHOP or in general with help from known scientific literature.

## Competing interests

The authors declare that they have no competing interests.

## Authors' contributions

AM carried out assembly of data, data analysis and interpretation and drafted main parts of the manuscript. TH, PC and MS conceived of the project and participated in its design and coordination. BD and JR carried out the cell culture, multilineage assay, flow cytometry analysis and contributed to the collection and interpretation of microarray data. In addition, JR helped to draft the manuscript. All authors read and approved the final manuscript.

## Supplementary Material

Additional file 1 **Genes presenting increased expression values during adipogenesis of human MSC**. Candidate genes whose mean signal expression values were increased during adipogenic development of human MSC were selected with help of SiPaGene database. The mean values of early (day 1 - day 3) or late (day 3 - day 7 - day 17 - fat) adipogenesis candidate genes are given in comparison with day 0. Affymetrix gene IDs are organized incrementally, in combination with a SD-value (SD: Standard deviation). Using SiPaGene each of the five performed MSC comparisons are working automatically with four different t-tests (see Methods). Only the strongest p-value is included for each ID and the most significant change (corresponding comparison) is highlighted in the file. Click here for file

Additional file 2 **Genes presenting decreased expression values during adipogenesis of human MSC**. Candidate genes whose mean signal expression values were decreased during adipogenic development of human MSC were selected with help of SiPaGene database. The mean values of early (day 1 - day 3) or late (day 3 - day 7 - day 17 - fat) adipogenesis candidate genes are given in comparison with day 0. Affymetrix gene IDs are organized incrementally, in combination with a SD-value (SD: Standard deviation). Using SiPaGene each of the five performed MSC comparisons are working automatically with four different t-tests (see Methods). Only the strongest p-value is included for each ID and the most significant change (corresponding comparison) is highlighted in the file. Click here for file

Additional file 3 **Table S1 (increased genes) and Table S2 (decreased genes). Controlling of Affymetrix probe set annotation**. Control for the correct annotation of target sequences (which is not shown) for Affymetrix probe sets shown in the Additional file 1 (increased genes) and Additional file 2 (decreased genes). Target sequences were compared to entries of the RefSeq release 46 using BLAST http://blast.ncbi.nlm.nih.gov/Blast.cgi and Aligner (CodonCode Corporation). Most target sequences mapped with 100% match to the 3' end of the corresponding RefSeq entries for mRNAs. Click here for file
