# Supplementary material for: Differential gene expression profiling of human bone marrow-derived mesenchymal stem cells during adipogenic development
Source: BMC Genomics. 2011 Sep 24;12:461. doi: 10.1186/1471-2164-12-461 (PMC3222637; doi:10.1186/1471-2164-12-461)
Supplement: Additional file 1 — Genes presenting increased expression values during adipogenesis of human MSC. Candidate genes whose mean signal expression values were increased during adipogenic development of human MSC were selected with help of SiPaGene database. The mean values of early (day 1 - day 3) or late (day 3 - day 7 - day 17 - fat) adipogenesis candidate genes are given in comparison with day 0. Affymetrix gene IDs are organized incrementally, in combination with a SD-value (SD: Standard deviation). Using SiPaGene each of the five performed MSC comparisons are working automatically with four different t-tests (see Methods). Only the strongest p-value is included for each ID and the most significant change (corresponding comparison) is highlighted in the file. [file 1471-2164-12-461-S1.PDF]

Table S1. Genes with increased expression values during adipogenesis of human MSC.

Affymetrix Gene ID	Gene symbol Gene title	Mean signal values ± SD						p-values §
		Day 0	Day 1	Day 3	Day 7	Day 17	Fat	
1552519_at	**ACVR1C** Activin A recept., type IC	9,4 ±8,5	117,2 ±20,2	155,8 ±28,8	176,2 ±33,8	518,8 ±60,6	1160,2 ±962,2	1.75E-10
1554044_a_at	**MRAP** Melanocortin 2 receptor accessory protein	20,3 ±10,4	107,9 ±11,4	236,1 ±56,4	172,4 ±9,7	591,7 ±97,1	1744,7 ±1549,3	9.44E-11
1555037_a_at	**IDH1** Isocitrate dehydrogenase 1 (NADP+), soluble	940,7 ±34,6	2198,1 ±197,2	2445,2 ±400,9	2582,8 ±401,2	2664,1 ±400,4	2796,1 ±1340,2	1.11E-15
1556035_s_at	**ZNF207** Zinc finger protein 207	79,5 ±15,1	138,6 ±25,3	161,6 ±23,2	134 ±41,7	148,5 ±41,2	497,6 ±96,5	2.00E-15
1558964_at	**FAT3** FAT tumor suppressor homolog 3 (Drosophila)	27,1 ±2,2	112 ±73,3	147,9 ±33,4	136,4 ±57,5	62,9 ±25,9	11,5 ±8,8	1.62E-08
200650_s_at	**LDHA** Lact. dehydrogenase A	9773,7 ±862,3	15790,1 ±1432,7	12054,6 ±2622,8	12679,9 ±3231,2	9922,0 ±3421,8	7084,5 ±1219	3.81E-07
200804_at	**TEGT** Testis enhanced gene transcript (BAX inhibitor 1)	864,5 ±216,3	844,8 ±222	1356,8 ±357,7	1449,9 ±298,3	1400,6 ±79,9	2379,7 ±154,4	3.39E-09
200871_s_at	**PSAP** Prosaposin (variant Gaucher disease and variant metachromatic leukodystrophy)	2641,5 ±764,8	3762,7 ±481,3	4993,8 ±538,1	3280,5 ±426,7	2409,5 ±312,3	9475,7 ±2265,9	1.66E-07
200880_at	**DNAJA1** DnaJ (Hsp40) homolog, subfamily A, member 1	783,7 ±99,5	1231,2 ±222,9	1945,2 ±380,5	1825,9 ±295,3	1217,1 ±170,9	696,3 ±113,6	6.40E-10
201060_x_at	**STOM** Stomatin	802,2 ±11,7	6227 ±76,3	4114,6 ±562,7	3892,4 ±476,6	3426,8 ±500,2	2136 ±265,7	1.38E-16

201395_at	**RBM5** RNA binding motif protein 5	320,6 ±33,9	362,9 ±27,2	418,7 ±48,6	419,6 ±36,3	243,6 ±12	1040,1 ±55,2	4.54E-11
201432_at	**CAT** Catalase	871,7 ±288,9	1727,6 ±467,1	1977,7 ±224,2	3036,8 ±415,6	4127,4 ±158,7	6431,2 ±4471,1	1.46E-09
201625_s_at	**INSIG1** Insulin induced gene 1	220,4 ±54,8	372,4 ±76,8	370,6 ±126,7	471,3 ±127,4	903,6 ±204,5	141,3 ±12,1	3.02E-10
202127_at	**PRPF4B** PRP4 pre-mRNA processing factor 4 homolog B (yeast)	80,3 ±10,1	136 ±9,3	163,6 ±8	129,8 ±23,7	93 ±53,4	292,7 ±28,3	1.77E-11
202350_s_at	**MATN2** Matrilin 2	119,1 ±46,1	219 ±34,5	418,5 ±120,8	232,7 ±63,2	337,7 ±88,2	869,3 ±541,2	3.75E-07
202449_s_at	**RXRA** Retinoid X receptor, alpha	436,5 ±109,5	1027,7 ±103,4	1599 ±235,1	2281,4 ±454,5	1695,3 ±82,1	981,8 ±317,7	2.23E-12
202605_at	**GUSB** Glucuronidase, beta	293 ±39,3	409 ±54	628,2 ±17,9	551,9 ±51,1	436,1 ±75,8	965,6 ±102,8	4.76E-09
202934_at	**HK2** Hexokinase 2	193,2 ±62,7	574,1 ±12,8	826,1 ±26,4	1822,2 ±525,4	1670,7 ±402,8	1075 ±646,7	2.91E-11
202992_at	**C7** Complem. component 7	26,1 ±33,6	11,7 ±17,1	44,9 ±34,9	150,8 ±54,8	277,6 ±8,1	400,6 ±296	4.23E-05
203296_s_at	**ATP1A2** ATPase, Na+/K+ transporting, alpha 2 (+) polypeptide	6,3 ±4,4	9,1 ±8,8	11,2 ±6,2	64,6 ±7,1	159,2 ±24,4	179,2 ±127,2	2.59E-09
203382_s_at	**APOE** Apolipoprotein E	20,0 ±12,7	44,2 ±27,7	670,5 ±553,5	2504,1 ±1779	2137,1 ±478,9	730,2 ±123	3.08E-10
203407_at	**PPL** Periplakin	34,3 ±45,4	76,0 ±26,8	294,5 ±177,2	502,1 ±56,3	402,7 ±48	596,5 ±484,3	2.64E-06
203424_s_at	**IGFBP5** Insulin-like growth factor binding protein 5	37,6 ±24,1	28,7 ±19,2	418,2 ±81,1	189,7 ±27,7	93,5 ±42	63,2 ±3	5.06E-07
203548_s_at	**LPL** Lipoprotein lipase	22,0 ±17,6	35,9 ±20	75,9 ±16	445 ±152,3	4008,2 ±700,4	7328,1 ±6143,4	2.32E-09
203627_at	**IGF1R** Insulin-like growth factor receptor	360,4 ±87,3	1047,6 ±240,5	298,6 ±52,5	339,3 ±22,5	303,8 ±94,9	88,5 ±10,4	4.17E-10

203980_at	**FABP4** Fatty acid binding protein 4, adipocyte	14,8 ±7,3	29,5 ±21,8	813,5 ±746,7	5371,5 ±1617,7	9578,2 ±1306,2	10378,4 ±8010,3	1.74E-13
204039_at	**CEBPA** CCAAT/enhancer binding protein (C/EBP), alpha	11,4 ±8,9	195,7 ±50,4	269,5 ±110	459 ±177,3	912,7 ±47,5	2841,2 ±2081,4	7.44E-12
204894_s_at	**AOC** Amine oxidase, copper containing 3 (vascular adhesion protein 1)	40,1 ±6,9	89,6 ±28,8	337,0 ±58,5	905,5 ±106,9	2151,8 ±561,6	5062,4 ±4341,2	9.08E-14
204997_at	**GPD1** Glycerol-3-phosphate dehydrogenase 1 (soluble)	47,9 ±13,7	38,7 ±34	43,9 ±17,6	53,6 ±34,3	319,7 ±147,5	1939,8 ±1801,3	4.77E-08
205204_at	**NMB** Neuromedin B	302 ±75,9	243,3 ±14,9	497,1 ±83,3	825,0 ±143,3	1402,3 ±779,4	1374,1 ±1041,3	5.69E-09
205498_at	**GHR** Growth hormone receptor	138,4 ±35,9	244,2 ±52,3	503,4 ±139,1	604,3 ±124,3	938,2 ±101,4	2088,8 ±1764,4	5.89E-12
205913_at	**PLIN** Perilipin	5,3 ±4,4	46,7 ±17,1	1517 ±207,7	1835 ±154	2569,6 ±479,5	5407,1 ±4663,6	8.44E-11
207175_at	**ADIPOQ** Adiponectin, C1Q and collagen domain containing	29,8 ±3,6	13,7 ±6,6	143,5 ±83,1	1312,4 ±313,7	4408,5 ±677,9	4556,4 ±4032,2	1.49E-20
207703_at	**NLGN4Y** Neuroligin 4, Y-linked	180,7 ±32,7	322,8 ±47,5	382,9 ±21,4	380,7 ±31,4	224,2 ±53,5	28.0 ±28,7	4.76E-10
208016_s_at	**AGTR1** Angiotensin II receptor, type 1	44,7 ±8,7	110,7 ±36,1	210,9 ±55,1	217,7 ±49,8	396,5 ±76,2	249,2 ±162,2	9.05E-11
208510_s_at	**PPARG** Peroxisome proliferative activated receptor, gamma	37,2 ±39,3	127,3 ±31,3	182,4 ±60,3	235,4 ±48,3	447 ±102	1131,6 ±977,6	1.35E-05
208949_s_at	**LGALS3** Lectin, galactoside-binding, soluble, 3 (galectin 3) /// galectin-3 internal gene	4796,9 ±540,7	4867 ±916,1	5543,6 ±1402,6	6385,4 ±1249,1	4403 ±652,2	7573,8 ±606,3	1.04E-11
209540_at	**IGF1** Insulin-like growth factor 1 (somatomedin C)	69,8 ±10,5	564,9 ±215,2	814,2 ±295,7	672,7 ±258,4	880,7 ±165,7	791,6 ±231,1	6.73E-14

209616_s_at	**CES1** Carboxylesterase 1 (monocyte/macrophage serine esterase 1)	56,7 ±23,1	47,6 ±22,2	159,6 ±92,6	436,4 ±86,4	1029,5 ±591,5	5946,9 ±5377	1.55E-10
211162_x_at	**SCD** Stearoyl-CoA desaturase (delta-9-desaturase)	98,4 ±46,3	77,1 ±49,5	120,2 ±88,3	410,5 ±52,5	2543,6 ±792	2920,4 ±2867,3	1.10E-12
211454_x_at	**FKSG49** FKSG49	155,1 ±10,9	186,4 ±36,1	239,4 ±45,2	284,5 ±74	303,9 ±140,3	420,8 ±110	1.18E-06
211569_s_at	**HADHSC** L-3-hydroxyacyl-Coenzyme A dehydrogenase, short chain	143,8 ±40,8	200,1 ±39,6	252,9 ±81,7	292,2 ±122,7	690,8 ±117,4	1057,1 ±786,8	2.48E-11
212135_s_at	**ATP2B4** ATPase, Ca++ transporting, plasma membrane 4	623,4 ±98,3	592,1 ±136,4	1178,8 ±260,6	1666,6 ±26,4	1505,5 ±243,9	1658,3 ±619,3	6.66E-16
212218_s_at	**FASN** Fatty acid synthase	93,0 ±38,8	253,7 ±76,3	299,7 ±175,1	264,6 ±153,8	721 ±82,2	843,7 ±608,1	3.18E-11
212510_at	**GPD1L** Gycerol-3-phosphate dehydrogenase 1-like	156,9 ±22,1	672,0 ±187,4	1898,9 ±475,2	1684,4 ±384,4	1463,9 ±380,2	348,0 ±185,7	4.44E-16
212793_at	**DAAM2** Dishevelled associated activator of morphogenesis 2	225 ±41,1	396,3 ±78,9	852 ±212,7	1070,4 ±105	1306 ±78,3	274,8 ±154,5	9.62E-13
213236_at	**SASH1** SAM and SH3 domain containing 1	244,2 ±58,2	641,9 ±67	572,1 ±132	484,8 ±150,9	596,7 ±131,8	1312,2 ±711,3	1.65E-07
213436_at	**CNR1** Cannabinoid receptor 1 (brain)	24,6 ±2,1	34,8 ±9,2	53,0 ±11,9	117,8 ±26,5	214,7 ±19,4	107,5 ±62,4	1.25E-09
213517_at	**PCBP2** Poly(rC) binding prot. 2	88,2 ±7,4	207,8 ±32,4	268,5 ±51,9	111 ±30	131,9 ±48,2	296,6 ±30,4	1.52E-11
214721_x_at	**CDC42EP4** CDC42 effector protein (Rho GTPase binding) 4	173 ±60,5	300,9 ±44	546,7 ±215,5	505,4 ±164,7	440,6 ±56,1	455,7 ±234,4	7.44E-07
217122_s_at	**SLC35E2** Solute carrier family 35, member E2	570 ±18,7	930,8 ±186,2	868,2 ±87,5	865 ±76,4	702,6 ±131,9	1149,7 ±26,7	1.18E-10

217882_at	**TMEM111** Transmemb. protein 111	894,6 ±105,4	1681,9 ±92,5	2783,6 ±234,2	2060,1 ±254,8	2013,5 ±732,3	1639,1 ±610,5	2.00E-15
218245_at	**LRRC54** Leucine rich rep. containing 54	238,1 ±66,7	576,3 ±245,6	1013,8 ±519,9	798,5 ±252,3	1080,7 ±108,7	600,5 ±301,1	1.75E-10
218346_s_at	**SESN1** Sestrin 1	170,0 ±43,8	1187,8 ±193,3	1433,1 ±519,5	622,8 ±113,6	569,4 ±189	556,7 ±92,4	3.22E-09
218975_at	**COL5A3** Collagen, type V, alpha 3	23,3 ±8,4	52,6 ±55,4	198,0 ±134,8	159,3 ±33,9	229,2 ±24,9	217,5 ±136,6	2.09E-10
219398_at	**CIDEC** Cell death-inducing DFFA-like effector c	80,4 ±25,9	130,1 ±10,6	265,4 ±77,1	327,8 ±27	1647,2 ±284,8	5183,3 ±4396,3	1.52E-09
219547_at	**COX15** COX15 homolog, cytochrome c oxidase assembly protein (yeast)	190 ±7,6	324,1 ±52,6	497,8 ±30,9	526,2 ±101,1	386,1 ±28,3	407,6 ±63,1	2.46E-14
219697_at	**HS3ST2** Heparan sulfate (glucosamine) 3-O-sulfotransferase 2	39,1 ±27,6	119,6 ±4,7	313,1 ±31,7	392,4 ±112	838,9 ±317,5	89,4 ±45,4	8.54E-09
219716_at	**APOL6** Apolipoprotein L, 6	101,4 ±6,4	105,9 ±23,9	121,3 ±34,6	222,5 ±92	303,7 ±78,2	256,9 ±65,9	3.94E-13
219761_at	**CLEC1A** C-type lectin domain family 1, member A	13,6 ±9,1	42,5 ±39,5	39,1 ±58,8	45,0 ±50,5	71,9 ±57,1	105,0 ±25,1	5.22E-05
220975_s_at	**C1QTNF1** C1q and tumor necrosis factor related protein 1	144,4 ±30,2	653,2 ±509,3	1044,6 ±862,8	807,4 ±630,3	1648,3 ±389,4	435,7 ±101,8	1.76E-11
221139_s_at	**CSAD** Cysteine sulfinic acid decarboxylase	50,0 ±19,6	98,5 ±24,8	239,6 ±56,1	185,3 ±52,3	211 ±58,6	482,1 ±228,8	2.94E-08
222750_s_at	**SRD5A2L** Steroid 5 alpha-reductase 2-like	240,2 ±78,1	349,3 ±136,2	567,5 ±136,4	586,1 ±177,4	881,3 ±95,7	318,1 ±112,5	8.37E-11
222853_at	**FLRT3** Fibronectin leucine rich transmembrane protein 3	89,3 ±15	180,2 ±54,5	301,8 ±135,1	260,8 ±41,1	265,2 ±74,8	31,0 ±15,4	1.25E-08

223130_s_at	**MYLIP**<br>Myosin regulatory light chain interacting protein	78,6 ±24,6	189,9 ±50,5	209 ±21,6	206,1 ±46,2	256,5 ±65,1	647 ±99,5	2.02E-10
223412_at	**KBTBD7**<br>Kelch repeat and BTB (POZ) domain cont. 7	155,8 ±18,3	204,8 ±36,1	330,7 ±74,8	337 ±68,7	202,1 ±31,2	27,0 ±6,8	1.37E-07
226038_at	**LONRF1**<br>LON peptidase N-terminal domain and ring finger 1	161,5 ±20,1	537 ±99,3	492 ±195,3	260,9 ±44,7	248 ±47,4	201,4 ±47,4	3.49E-12
226064_s_at	**DGAT2**<br>Diacylglycerol O-acyltransferase homolog 2 (mouse)	30,5 ±38,7	45,8 ±31,3	30,8 ±28,4	121,6 ±97,4	548,5 ±266,1	4211 ±3630,9	1.12E-04
226509_at	**ZNF641**<br>Zinc finger protein 641	163,3 ±8	187,3 ±30,6	235,3 ±25,6	251,5 ±23,8	220 ±53	384 ±29,6	4.97E-14
226547_at	**MYST3**<br>Histone acetyltransferase 3	203,8 ±33	282,7 ±57,7	374,5 ±14,7	356,1 ±13,5	252,8 ±45,4	559,3 ±112,7	6.52E-11
226568_at	**FAM102B**<br>Family with sequence similarity 102, member B	132,5 ±36,2	166,9 ±13	184,6 ±16	180,4 ±28,7	148,5 ±47,2	338,1 ±51,7	2.17E-09
226576_at	**ARHGAP26**<br>Rho GTPase activating protein 26	23,3 ±11,9	31,4 ±28,3	63,4 ±23,4	87,0 ±7,2	131,5 ±65	74,5 ±88,4	3.80E-05
227899_at	**VIT**<br>Vitrin	69,6 ±44,3	81,8 ±88,6	336,9 ±156,9	336,5 ±124,1	566,9 ±28	365,5 ±146,8	6.62E-08
228410_at	**GAB3**<br>GRB2-associated binding protein 3	4,2 ±1,8	10,5 ±1,9	7,3 ±3,3	6,3 ±0,7	32 ±19,4	148,8 ±56,4	1.04E-08
229487_at	**EBF1**<br>Early B-cell factor 1	168,7 ±10,9	594,9 ±129,1	831,9 ±20,2	1026,9 ±164,7	682,9 ±188,8	808,9 ±578	1.80E-14
229839_at	**SCARA5**<br>Scavenger receptor class A, member 5 (putative)	5,7 ±2,4	32,8 ±17	351,4 ±142,1	821,5 ±197,9	1447,7 ±693	1973,2 ±1257,3	8.72E-13
230180_at	**DDX17**<br>DEAD (Asp-Glu-Ala-Asp) box polypeptide 17	118,8 ±48,1	177,1 ±24,6	276,1 ±112,9	230,3 ±124,5	136,5 ±121,9	303,6 ±153,8	1.28E-05

235306_at	**GIMAP8**<br>GTPase, IMAP family member 8	16 ±7,7	41,9 ±50,6	34 ±32,7	33,2 ±19,7	85 ±58,7	626,4 ±62,6	7.82E-12
235956_at	**KIAA1377**<br>KIAA1377, unknown function	100,4 ±24	147,7 ±28,3	305,9 ±26,9	283,1 ±60,9	179,9 ±36,2	339,6 ±121,3	8.50E-09
236361_at	**GALNTL2**<br>UDP-N-acetyl-alpha-D-galactosamine:polypeptide N-acetylgalactos-aminyltransferase-like 2	152,6 ±102,7	3934,9 ±313,8	1576 ±362,6	1474,7 ±717	897,9 ±389,6	937,5 ±215,8	1.27E-10
237475_x_at	**SEPP1**<br>Selenoprotein P1, plasma	124,8 ±24,1	210 ±35,1	251,6 ±91,4	171 ±59,2	146,8 ±37,3	960,2 ±372,6	1.14E-11
239629_at	**CFLAR**<br>CASP8 and FADD-like apoptosis regulator	58,8 ±38,6	76,1 ±49,8	134,0 ±60,1	154,9 ±89	82,7 ±60,7	1039,1 ±97,1	4.55E-09
242738_s_at	**ZFHX3**<br>Zinc finger homeodomain protein 3	168,4 ±12,6	402,3 ±97,4	497,9 ±179,4	318,8 ±104,8	279 ±43,5	296,3 ±23,3	2.36E-09
244766_at	**SMG1**<br>PI-3-kinase-related kinase	26,1 ±4,8	56,8 ±33,2	32,2 ±9,1	41,8 ±11,1	50,8 ±39,4	96,8 ±14,6	6.60E-11
43427_at	**ACACB**<br>Acetyl-Coenzyme A carboxylase beta	18,9 ±21,6	217,3 ±70,6	574,8 ±119,4	425,6 ±131,4	670,6 ±242,8	1309,9 ±1170,5	1.82E-07

§ The most significant change from which the p-value was taken is highlighted for each Gene ID.
