# Supplementary material for: Differential gene expression profiling of human bone marrow-derived mesenchymal stem cells during adipogenic development
Source: BMC Genomics. 2011 Sep 24;12:461. doi: 10.1186/1471-2164-12-461 (PMC3222637; doi:10.1186/1471-2164-12-461)
Supplement: Additional file 2 — Genes presenting decreased expression values during adipogenesis of human MSC. Candidate genes whose mean signal expression values were decreased during adipogenic development of human MSC were selected with help of SiPaGene database. The mean values of early (day 1 - day 3) or late (day 3 - day 7 - day 17 - fat) adipogenesis candidate genes are given in comparison with day 0. Affymetrix gene IDs are organized incrementally, in combination with a SD-value (SD: Standard deviation). Using SiPaGene each of the five performed MSC comparisons are working automatically with four different t-tests (see Methods). Only the strongest p-value is included for each ID and the most significant change (corresponding comparison) is highlighted in the file. [file 1471-2164-12-461-S2.PDF]

Table S1. Genes with decreased expression values during adipogenesis of human MSC.

Affymetrix Gene ID	Gene symbol / Gene title	Mean signal values ± SD						p-value §
		Day 0	Day 1	Day 3	Day 7	Day 17	Fat	
1558105_a_at	--------------- Homo sapiens, Similar to LOC169932, clone IMAGE: 4499203	195,5 ±46,9	136,9 ±20,7	119,6 ±38,8	51,3 ±13	28,0 ±12,5	51,4 ±33	4.53E-07
200014_s_at	**HNRPC** Heterogeneous nuclear ribonucleoprotein C (C1/C2)	1997,2 ±277,3	1533,4 ±327,2	1838,2 ±300,5	1622,1 ±250,3	1310,3 ±116,4	1004,7 ±180,1	2.09E-06
200606_at	**DSP** Desmoplakin	1591,4 ±345	940,1 ±244,5	388,7 ±70,5	501,4 ±191,7	238,5 ±78,7	71,5 ±40,5	2.17E-10
200654_at	**P4HB** Procollagen-proline, 2-oxoglutarate 4-dioxygenase	6967,7 ±854,6	7155,3 ±596,9	5269,9 ±465,1	3724,4 ±869,2	5697,1 ±1135	3397,9 ±1090,5	1.93E-05
200663_at	**CD63** CD63 molecule	8394,1 ±1122,1	8116,5 ±1756,6	5951,8 ±1081,2	5044,8 ±626,2	6550,3 ±783,6	8283,0 ±1054,4	1.14E-06
200700_s_at	**KDELR2** KDEL (Lys-Asp-Glu-Leu) endoplasmic retic. protein retention receptor 2	5648,7 ±742,6	3679,4 ±781,4	2659,2 ±874,5	3975,6 ±1109,6	3355,9 ±468	1085,2 ±160,3	4.70E-13
200704_at	**LITAF** Lipopolysaccharide-induced TNF factor	598,0 ±39,6	605,1 ±161,5	339,3 ±86,5	231,1 ±82,9	161,4 ±47,2	1853,5 ±571,5	1.72E-10
200734_s_at	**ARF3** ADP-ribosylation factor 3	1269,4 ±146,1	830,3 ±65,7	524,8 ±92,1	726,7 ±153,7	714,9 ±107,7	677,1 ±237,8	3.49E-07
200745_s_at	**GNB1** Guanine nucleotide binding protein, beta polypeptide 1	3468,2 ±229,8	1415,6 ±449	1224,7 ±364,8	1890,8 ±553,8	1286,3 ±222,6	1610,0 ±88,3	1.57E-12
200757_s_at	**CALU** Calumenin	3255,2 ±654,8	2597,9 ±616,9	1450,5 ±184,5	1953,8 ±223,7	2477,8 ±1048	789,4 ±165,1	9.63E-10
200816_s_at	**PAFAH1B1** Platelet-activating factor acetylhydrolase, isof. Ib	1101,7 ±211,4	298,3 ±72,1	672,6 ±98	800,1 ±162,3	494 ±76,4	997,7 ±267,1	3.63E-06

200891_s_at	**SSR1** Signal sequence receptor, alpha (translocon-associated protein alpha)	7134,6 ±1766	4761,5 ±1213,9	5226,0 ±1404,2	5716,0 ±1574,2	4886,7 ±1726,3	3155,3 ±874,9	2.68E-07
200902_at	**SEP15** 15 kDa Selenoprotein	3614,4 ±561,6	2989,9 ±156,5	2702,7 ±358,5	3166,3 ±359,7	2242,3 ±339,6	1964,8 ±354,1	1.34E-07
200989_at	**HIF1A** Hypoxia-inducible factor 1, alpha	10599,6 ±1098	10715,8 ±2192,8	6797,0 ±2163,8	7055,3 ±1489,7	4804,4 ±1157,3	2266,1 ±1091,5	4.04E-07
200998_s_at	**CKAP4** cytoskeleton-associated protein 4	3684,4 ±321,8	1872,8 ±159,6	1154,0 ±315,5	1788,8 ±562,9	1644,0 ±254,8	402,0 ±248,1	1.14E-09
201097_s_at	**ARF4** ADP-ribosylation factor 4	8264,5 ±780	3695,6 ±940,9	3629,7 ±648,4	5190,4 ±705,6	4765,8 ±1039,5	3611,9 ±219,1	3.80E-14
201099_at	**USP9X** ubiquitin specific peptidase 9, X-linked	599,0 ±112,7	378,2 ±24,3	420,1 ±52,7	430,1 ±88,1	265,4 ±19,6	615,1 ±64,6	5.34E-07
201148_s_at	**TIMP3** Tissue inhibitor of metalloproteinases 3	2856,8 ±367,7	1298,4 ±234,6	1208,5 ±162,7	1785,8 ±682,5	834,8 ±412,8	1292,4 ±452,3	3.53E-07
201162_at	**IGFBP7** Insulin-like growth factor binding protein 7	14114,2 ±833,6	13902,7 ±1940,2	13576,5 ±1302	10077,8 ±1407,7	8979,1 ±1563,1	5139,8 ±417,8	2.22E-16
201261_x_at	**BGN** Biglycan	2417,9 ±23,5	2351,4 ±669,6	2434,8 ±797,3	786,3 ±264,2	563,1 ±120,5	573,3 ±449,7	2.44E-15
201278_at	**DAB2** Disabled homolog 2, mitogen-responsive phosphoprotein	1753,7 ±254,7	714,7 ±329,8	427,9 ±167	743,9 ±257,6	505,8 ±142,4	1785,5 ±763,7	1.72E-10
201286_at	**SDC1** Syndecan 1	408,5 ±36,5	269,3 ±86,1	208,7 ±92,1	85,5 ±29,9	112,0 ±25,3	68,8 ±88,6	1.36E-08
201330_at	**RARS** Arginyl-tRNA synthetase	1941,4 ±250,5	1180,5 ±197,6	1380,5 ±556,4	1382,4 ±354,4	1331,1 ±687	646,3 ±153,2	2.20E-06
201462_at	**SCRN1** Secernin 1	804,7 ±97,3	408,6 ±59,9	386,0 ±36,7	470,8 ±37,7	387,4 ±81,4	283,9 ±69,7	4.44E-10
201470_at	**GSTO1** Glutathione S-transferase omega 1	2906,4 ±450,4	2487,9 ±557,9	1656,2 ±230,9	1958,0 ±428,5	1432,5 ±196,5	2037,0 ±1535,7	2.41E-08

201560_at	**CLIC4** Chloride intracellular channel 4	7942,4 ±386,5	7795,8 ±1816,4	6230,7 ±1777,5	6411,2 ±1155,8	3842,4 ±1257,3	2779 ±259	2.22E-16
201594_s_at	**PPP4R1** Protein phosphatase 4, regulatory subunit 1	783,0 ±122,5	537,3 ±96,5	371,9 ±5,9	397,6 ±48,8	407,0 ±15,8	526,5 ±79,7	3.12E-09
201719_s_at	**EPB41L2** Erythrocyte membrane protein band 4.1-like 2	365,8 ±56,1	324,5 ±33,3	261,9 ±56,6	217,4 ±38,2	87,4 ±21	1350,4 ±444,4	4.10E-09
201994_at	**MORF4L2** Mortality factor 4 like 2	8304,8 ±800,1	10558 ±524,2	9066,3 ±1619,6	9449,8 ±1393,4	6968,1 ±1463,5	2899,4 ±150,8	1.24E-14
202052_s_at	**RAI14** Retinoic acid induced 14	2087,2 ±156,5	553,2 ±193,6	591,9 ±316	603,6 ±196,2	384,7 ±121,7	188,6 ±137,1	1.51E-10
202074_s_at	**OPTN** Optineurin	1125,8 ±154,3	763,2 ±53,6	522,4 ±57,7	489,0 ±69,6	566,7 ±165,8	885,7 ±238,5	1.21E-08
202369_s_at	**TRAM2** Translocation associated membrane protein 2	1473,9 ±253,8	1596,4 ±178,9	780,5 ±170,2	1255,2 ±244,6	1127,5 ±449	829,1 ±68,9	4.17E-09
202418_at	**YIF1A** Yip1 interacting factor homolog A (S. cerevisiae)	1832,8 ±511,5	534 ±17,9	436,5 ±100,4	855,3 ±81,4	957,9 ±218,9	565 ±66,7	8.88E-16
202591_s_at	**SSBP1** Single-stranded DNA binding protein 1	2443,5 ±263,9	1263,5 ±205,9	1320,1 ±308,5	1641,8 ±198,9	1585,3 ±155,4	1411,5 ±165,5	1.83E-09
202619_s_at	**PLOD2** Procollagen-lysine, 2-oxoglutarate 5-dioxygenase 2	3635,2 ±1061,6	1438,7 ±147,8	1561 ±197,6	1880,6 ±357,1	3269,8 ±1682,3	1041,2 ±358,3	1.22E-08
202669_s_at	**EFNB2** Ephrin-B2	641,8 ±114,3	442,4 ±25	370,8 ±90,5	160,1 ±40,1	209,8 ±76,7	168 ±69,7	2.82E-08
202693_s_at	**STK17A** Serine / threonine kinase 17a (apoptosis-inducing)	2375,1 ±183,2	1475,5 ±582,3	1564,8 ±374,3	1270 ±431,9	911,6 ±227,9	524,6 ±81,7	5.07E-13
202696_at	**OXSR1** Oxidative-stress resp. 1	534,8 ±57,8	340,9 ±104,1	284,5 ±58,5	396,6 ±133,4	304,0 ±81,1	454,3 ±101,1	9.34E-08
202722_s_at	**GFPT1** Glutamine-fructose-6-phosp. transaminase 1	1215,8 ±116,7	433,2 ±83,7	390,8 ±76,7	778,4 ±61,7	790,2 ±151,2	563,2 ±267,2	1.00E-06

202986_at	**ARNT2**<br>Aryl-hydrocarbon receptor nuclear translocator 2	286,2 ±79,7	113,6 ±53,9	109,6 ±7,7	65,1 ±27,1	41,1 ±14,2	14,2 ±12,9	3.88E-09
203020_at	**RABGAP1L**<br>RAB GTPase activating protein 1-like	171,9 ±21,9	142,0 ±42,1	102,6 ±21	82,8 ±18,1	60,0 ±40,8	375,7 ±59,3	7.27E-09
203072_at	**MYO1E**<br>Myosin IE	354,8 ±48	270,9 ±25,9	191,1 ±46,8	151,0 ±52,6	174,8 ±66	103,4 ±47,9	8.90E-11
203083_at	**THBS2**<br>Thrombospondin 2	7304,1 ±1603,1	4314,4 ±1517,8	1798,4 ±1342,3	2470,2 ±1203,6	2759,3 ±1554,4	662,8 ±251,8	3.82E-14
203201_at	**PMM2**<br>Phosphomannomutase 2	390,4 ±77,8	84,9 ±16,5	99,0 ±15,1	143,4 ±52,8	148,1 ±31,3	125,6 ±38,4	4.58E-07
203349_s_at	**ETV5**<br>Ets variant gene 5 (ets-related molecule)	352,7 ±57,3	218,9 ±49,6	221,3 ±42,7	99,4 ±8,6	123,5 ±61,9	141,8 ±65,6	6.38E-05
203404_at	**ARMCX2**<br>Armadillo repeat containing, X-linked 2	2763,8 ±481,7	1544,1 ±302,9	1714,8 ±475,5	2015,7 ±549,5	1269,2 ±430,4	432,9 ±176,7	9.59E-13
203510_at	**MET**<br>Met proto-oncogene	2114,6 ±954,6	1027,4 ±526,4	1014,9 ±487,4	760,7 ±240,9	314,0 ±142,3	379,1 ±139,7	1.00E-08
203620_s_at	**FCHSD2**<br>FCH and double SH3 domains 2	394,2 ±40	376,7 ±97,3	224,3 ±62,6	180,3 ±23,2	156,1 ±15	301,7 ±85,3	8.35E-08
203675_at	**NUCB2**<br>Nucleobindin 2	1157,8 ±81	977,7 ±84,4	707,7 ±66,2	580,4 ±91,2	452,9 ±32,1	581,1 ±262,1	2.06E-09
203810_at	**DNAJB4**<br>DnaJ (Hsp40) homolog, subfamily B, member 4	2026,1 ±227,3	646,6 ±80,7	1238,9 ±167,4	1226,7 ±99	1148,3 ±461,1	441,0 ±210,8	5.78E-11
203939_at	**NT5E**<br>5’-nucleotidase, ecto (CD73)	1461,7 ±395	458,9 ±61,3	424,4 ±64	346,5 ±45,5	282,7 ±36,3	405,6 ±139	8.88E-16
204004_at	**PAWR**<br>PRKC, apoptosis, WT1, regulator	1730,3 ±216,7	612,7 ±109,5	501,6 ±65,5	795,4 ±212,9	620,3 ±207,6	227,1 ±131,6	1.89E-10
204017_at	**KDELR3**<br>KDEL (Lys-Asp-Glu-Leu) endoplasmic reticulum protein retention receptor 3	5596,3 ±515,6	1646,5 ±368	783,5 ±124,7	1745,7 ±560,2	1320,1 ±135,7	757,7 ±131,5	7.33E-15

204671_s_at	**ANKRD6**<br>Ankyrin repeat domain 6	306,4<br>±32,5	191<br>±39,8	73,1<br>±14,9	59,0<br>±21,4	44,4<br>±30	86,5<br>±38,8	1.37E-13
206298_at	**RhoGAP22**<br>Rho GTPase activating protein 22	221,5<br>±47,4	17,4<br>±8,7	10,3<br>±4,8	29,7<br>±32,6	32,8<br>±20,7	42,2<br>±38	4.69E-12
206580_s_at	**EFEMP2**<br>EGF-containing fibulin-like extracellular matrix protein 2	2465,2<br>±538,6	2232,8<br>±356,2	1497,8<br>±304,7	805,1<br>±187,3	972,3<br>±63,7	521,4<br>±72,2	1.57E-12
207358_x_at	**MACF1**<br>Microtubule-actin crosslinking factor 1	835,4<br>±93,9	433,2<br>±116,4	284,2<br>±53,2	300,3<br>±15,4	267,9<br>±125,5	593,9<br>±120,4	1.69E-10
209094_at	**DDAH1**<br>Dimethylarginine dimethylaminohydrolase 1	6011,7<br>±651,3	3174,8<br>±1763,1	2153,5<br>±420,7	3642,7<br>±1344,3	2388,4<br>±357,6	333,3<br>±59,4	6.12E-13
209526_s_at	**HDGFRP3**<br>Hepatoma-derived growth factor, related protein 3	761,0<br>±115,1	570,8<br>±41,2	412,8<br>±77,4	416,8<br>±19,4	457,7<br>±120,9	238,5<br>±28	8.88E-16
209578_s_at	**POFUT2**<br>Prot. O-fucosyltransfer. 2	506,4<br>±105,2	360,2<br>±140,3	311,5<br>±69,5	313,7<br>±98,4	204,2<br>±118,8	137,4<br>±20,5	1.44E-10
211071_s_at	**MLLT11**<br>Myeloid/lymphoid or mixed-lineage leukaemia	1975,4<br>±84,8	912,5<br>±42,7	783,3<br>±177,1	666,5<br>±88	622,3<br>±176,1	84,5<br>±37,9	3.75E-11
211202_s_at	**JARID1B**<br>Jumonji, AT rich interactive domain 1B (RBP2-like)	852,4<br>±183,8	728,9<br>±222,3	519,6<br>±166,3	507,8<br>±156,3	349,9<br>±74,6	256,7<br>±20,5	1.34E-10
211325_x_at	**LOC171220**<br>Destrin-2 pseudogene	296,1<br>±128,1	277,5<br>±41,7	261,5<br>±16	295,0<br>±125,5	297,4<br>±120,6	120,5<br>±17,9	1.20E-11
211725_s_at	**BID**<br>BH3 interacting domain death agonist	272,3<br>±54,5	132,1<br>±78	106,3<br>±43,9	76,7<br>±52,1	202,4<br>±38,8	293,1<br>±188,7	2.70E-06
212256_at	**GALNT10**<br>UDP-GalNAc:polypeptide N-acetyl-galactosaminyl-transferase 10	1990,5<br>±182,9	1614,6<br>±261,3	714,5<br>±138,2	440,9<br>±76,2	420,8<br>±47,9	198,6<br>±100,7	3.33E-12
212344_at	**SULF1**<br>Sulfatase 1	4320,4<br>±501,7	3493<br>±191,6	1585,9<br>±588,9	1040,5<br>±621,1	191,4<br>±138,9	175,7<br>±51,9	5.35E-13

212565_at	**STK38L**<br>Serine/threonine kinase 38 like	529,1 ±84	170,2 ±52,1	225,9 ±18,1	184,1 ±23,9	152,5 ±27,8	302,9 ±29,5	1.35E-10
212822_at	**HEG1**<br>HEG homolog 1 (zebrafish)	754 ±156	511,3 ±125,6	218,1 ±59,8	234,4 ±51,5	185,7 ±184,7	402,2 ±176,7	2.30E-10
212848_s_at	**C9orf3**<br>Chromosome 9 open reading frame 3	763,4 ±207,3	295,3 ±135,9	200,3 ±119,4	259,7 ±141,2	136,3 ±77,2	333,7 ±92,9	4.00E-12
213059_at	**CREB3L1**<br>cAMP responsive element binding protein 3-like 1	928,9 ±163,2	275,1 ±121,9	2009 ±80,1	298,1 ±88,9	404,1 ±209,3	52,4 ±15,9	2.00E-15
213338_at	**TMEM158**<br>Transmembrane prot. 158	649,5 ±60,9	356,3 ±48,4	336,9 ±42,1	196,9 ±59,3	372,7 ±184,7	53,1 ±44,3	2.12E-08
213867_x_at	**ACTB**<br>Actin, beta	15863,8 ±1194,8	11161,8 ±622,1	8674,9 ±966,7	13619,2 ±2723,5	9947,5 ±2525,7	12716,5 ±3298	4.84E-10
214316_x_at	**CALR**<br>Calreticulin	587,4 ±163,3	551,2 ±89,1	499,6 ±151,3	501,6 ±148,3	316,2 ±149,1	106,4 ±52	1.79E-08
218189_s_at	**NANS**<br>N-acetylneuraminic acid synthase	814 ±13,4	637 ±111,9	358,8 ±52,5	334,8 ±62,4	364,1 ±87,9	404,3 ±102,4	1.39E-09
218775_s_at	**WWC2**<br>WW, C2 and coiled-coil domain containing 2	549,7 ±77,7	236,9 ±56	198,2 ±27,7	178,9 ±38	152 ±61	156,8 ±75,9	5.56E-13
218802_at	**CCDC109B**<br>Coiled-coil domain containing 109B	663,3 ±89,8	466,1 ±95,4	244,1 ±65,6	215,0 ±79,9	205,6 ±54,4	566,6 ±243,7	4.03E-10
218854_at	**SART2**<br>Squamous cell carcinoma antigen recognized by T cells 2	3187,7 ±583,4	2044 ±905,1	1871,4 ±781	1823,6 ±751,3	1488,3 ±432,6	1030,1 ±497,6	1.55E-09
219038_at	**MORC4**<br>MORC family CW-type zinc finger 4	407,6 ±75,6	103,9 ±40,6	101,6 ±32,6	132,3 ±35,2	149,0 ±23,4	266,7 ±54	4.30E-09
221007_s_at	**FIP1L1**<br>Factor interacting with PAP1 like 1 (S. cerevisiae)	330,6 ±77,7	261,5 ±22	286,2 ±63,7	333,4 ±55,9	192,3 ±32	139,7 ±18,3	1.13E-13
221031_s_at	**APOLD1**<br>Apolipoprotein L domain containing 1	84,7 ±12,4	93,7 ±22	73,1 ±11,4	63,5 ±23	58,2 ±30,7	292,4 ±10,7	1.58E-14

222634_s_at	**TBL1XR1** Transducin (beta)-like 1X-linked receptor 1	403,8 ±49,5	247,1 ±29,2	84,5 ±115,8	272 ±6,8	210,4 ±36,8	119,1 ±39,8	5.46E-11
222978_at	**SURF4** Surfeit 4	1989,3 ±113,6	1416,3 ±157,4	1147,3 ±74,4	1316,5 ±134,7	1290,2 ±318,6	1716,6 ±294,9	6.30E-08
223374_s_at	**B3GALNT1** Beta-1,3-N-acetylgalactosaminyl-transferase 1	259,1 ±66,5	157,5 ±39,7	124,1 ±26,8	116,0 ±17,1	120,8 ±31	74,9 ±11,9	5.47E-10
224560_at	**TIMP2** Tissue inhibitor of metalloproteinases 2	3208,8 ±85,1	3429,7 ±260,6	3322,3 ±103,6	1505,5 ±204,1	1056,6 ±145	2880,7 ±518,5	1.90E-13
224677_x_at	**C11orf31** Chromosome 11 open reading frame 31	1505,6 ±428,8	584,4 ±138,7	417,7 ±81	366,2 ±13,4	333,6 ±170,6	776,2 ±113,7	7.08E-10
224771_at	**NAV1** Nuron navigator 1	842,2 ±209,1	584,7 ±45,3	610,3 ±251,7	672,0 ±405,4	270,1 ±261,3	212,4 ±42,7	1.15E-11
224955_at	**TEAD1** TEA domain family member 1 (SV40 transcriptional enhancer activator factor)	2223,5 ±325,5	1582,1 ±237,7	1708,9 ±266,5	1305,5 ±243	882,2 ±70,4	1117,5 ±653,8	3.19E-10
225152_at	**ZNF622** Zinc finger protein 622	744,3 ±56,1	779,2 ±161	572,1 ±155,3	507,2 ±218,1	461,8 ±122,9	229 ±43,2	2.55E-08
225188_at	**RAPH1** Ras-associated and pleckstrin homology domains-containing protein 1	1463,9 ±364,8	405,4 ±234,2	366,9 ±43,2	338,6 ±120,1	226,2 ±54,6	675,6 ±336,3	1.20E-11
227280_s_at	**CCNYL1** Cyclin-Y-like protein 1	647 ±13,3	402,2 ±162,9	363 ±96,5	402,1 ±128	249,7 ±50,2	328,5 ±86,2	1.25E-08
227320_at	**FAM101A** Family with sequence similarity 101, member A	759,1 ±130,4	37,7 ±32,2	77,0 ±21,3	80,1 ±56	152,8 ±69,9	19,8 ±6,5	4.59E-12
227566_at	**NTM** Neurotrimin	1938,9 ±151,5	1017,4 ±486,7	629,5 ±229,5	705,3 ±453,9	166,7 ±26,9	541,6 ±399,7	7.58E-14
227638_at	**KIAA1632** KIAA1632	465,6 ±13	217,8 ±18,8	173,8 ±6	129,1 ±31,2	176,2 ±29,5	168,5 ±1,5	1.78E-15

228293_at	**DEPDC7**<br>DEP domain containing 7	301,3 ±42,5	145,3 ±41	152 ±25,1	150,8 ±5,7	99,3 ±17	24,7 ±5	4.44E-16
229555_at	**GALNT5**<br>UDP-N-acetyl-alpha-D-galactosamine:polypeptide N-acetylgalacto-saminyltransferase 5	711,3 ±98,4	614,4 ±178,9	221,3 ±9,6	238,3 ±13,9	245,4 ±117,4	86,5 ±95,5	1.40E-13
229582_at	**INO80 B**<br>INO80 complex subunit B	377,6 ±158,4	83,8 ±48,6	103,4 ±11,9	179,6 ±49,5	222 ±22,4	325,7 ±51,5	5.33E-08
231841_s_at	**KIAA1462**<br>KIAA1462	570,4 ±102,8	364,8 ±153,9	251,3 ±72,7	168,1 ±6	143,0 ±27,6	101,8 ±45,7	3.70E-12
231999_at	**ANKRD11**<br>Ankyrin repeat domain 11	316,5 ±38,7	313,7 ±88	259,7 ±114,8	245,4 ±103,5	153,1 ±92,1	46,3 ±9,5	2.35E-09
243864_at	**CCDC80**<br>Coiled-coil domain containing 80	504,7 ±30,3	297,4 ±47,3	89,8 ±12,8	150,3 ±1,8	170,0 ±36,3	283,8 ±116,6	8.33E-14

§ The most significant change from which the p-value was taken is highlighted for each Gene ID.
