# Supplementary material for: Differential gene expression profiling of human bone marrow-derived mesenchymal stem cells during adipogenic development
Source: BMC Genomics. 2011 Sep 24;12:461. doi: 10.1186/1471-2164-12-461 (PMC3222637; doi:10.1186/1471-2164-12-461)
Supplement: Additional file 3 — Table S1 (increased genes) and Table S2 (decreased genes). Controlling of Affymetrix probe set annotation. Control for the correct annotation of target sequences (which is not shown) for Affymetrix probe sets shown in the Additional file 1 (increased genes) and Additional file 2 (decreased genes). Target sequences were compared to entries of the RefSeq release 46 using BLAST http://blast.ncbi.nlm.nih.gov/Blast.cgi and Aligner (CodonCode Corporation). Most target sequences mapped with 100% match to the 3' end of the corresponding RefSeq entries for mRNAs. [file 1471-2164-12-461-S3.PDF]

Probeset_ID	Gene Symbol (increased genes)	RefSeq sequence Release 46	Comment
552519_at	ACVR1C	NM_145259.2	ok
1554044_a_at	MRAP	NM_178817.3	ok
1555037_a_at	IDH1	NM_005896	ok
1556035_s_at	ZNF207	GNOMON 16744323.m, AL834501.1	ok, ZNF207c
1558964_at	FAT3	NM_001008781.2	ok
200650_s_at	LDHA	NM_005566.3	ok
200804_at	TMBIM6	NM_003217.2	ok
200871_s_at	PSAP	NM_002778.2	target sequence is downstream of first polyA-site and upstream of other polyA-site(s)
200880_at	DNAJA1	GNOMON 10894623.m	ok
201060_x_at	STOM	NM_004099.4	ok
201395_at	RBM5	NM_005778.2	ok
201432_at	CAT	NM_001752.3	ok
201625_s_at	INSIG1	NM_005542.4	ok
202127_at	PRPF4B	NM_003913.4	target sequence is downstream of first polyA-site and upstream of other polyA-site(s)
202350_s_at	MATN2	NM_002380.3	ok
202449_s_at	RXRA	NM_002957.4	ok
202605_at	GUSB	NM_000181.3	ok
202934_at	HK2	NM_000189.4	ok
202992_at	C7	NM_000587.2	ok
203296_s_at	ATP1A2	NM_000702.3	ok
203382_s_at	APOE	NM_000041.2	ok
203407_at	PPL	NM_002705.4	ok
203424_s_at	IGFBP5	NM_000599.3	ok, target sequence is upstream of CDS within exon 1
203548_s_at	LPL	NM_000237.2	ok
203627_at	IGF1R	NM_000875.3	ok
203980_at	FABP4	NM_001442.2	ok
204039_at	CEBPA	NM_004364.3	ok
204894_s_at	AOC3	NM_003734.2	ok
204997_at	GPD1	NM_005276.2	ok
205204_at	NMB	NM_021077.3	ok
205498_at	GHR	NM_000163.2	oligo#11 of target sequence is downstream of polyA - site

205913_at	PLIN1	NM_002666.4	ok
207175_at	ADIPOQ	NM_001177800.1	ok
207703_at	NLGN4Y	NM_014893.4	target sequence is downstream of first polyA-site and upstream of other polyA-site(s)
208016_s_at	AGTR1	NM_000685.4	ok
208510_s_at	PPARG	NM_138712.3	ok
208949_s_at	LGALS3	NM_002306.3	ok
209540_at	IGF1	NM_001111283.1	ok
209616_s_at	CES1	NM_001025195.1	ok
211162_x_at	SCD	NM_005063.4	ok
211454_x_at	FKSG49	GNOMON 19020193, XM_003118496.1	BLAST without hit
211569_s_at	HADH	NM_004998.2	target sequence is downstream of first polyA-site and upstream of other polyA-site(s)
212135_s_at	ATP2B4	NM_003247.2	ok
212218_s_at	FASN	NM_000303.2	ok
212510_at	GPD1L	NM_004454.2	ok
212793_at	DAAM2	NM_014782.5	ok
213236_at	SASH1	NM_001127500.1	ok
213436_at	CNR1	NM_014824.2	ok
213517_at	PCBP2	NM_005013.2	target sequence does not match with any of the 7 variants of mRNA sequences; maps to intron sequence of PCBP2
214721_x_at	CDC42EP4	GNOMON 19430213.m, AB209922.1	ok
217122_s_at	SLC35E2	NM_002526.3	ok
217882_at	TMEM111	GNOMON 17696263.m	ok
218245_at	TSKU	NM_006855.2	ok
218346_s_at	SESN1	NM_014942.3	ok
218975_at	COL5A3	NM_021226.2	ok
219398_at	CIDEC	NM_016938.4	ok
219547_at	COX15	NM_012090.4	ok

219697_at	HS3ST2	NM_012137.3	first polyA-site within target sequence, excludes oligo #9-11, next polyA-sites downstream of target sequence
219716_at	APOL6	NM_016073.2	ok
219761_at	CLEC1A	NM_015227.4	ok
220975_s_at	C1QTNF1	NM_006818.3	ok
221139_s_at	CSAD	NM_006618.3	ok
222750_s_at	SRD5A3	NR_033796.1	ok
222853_at	FLRT3	NM_197966.1	ok
223130_s_at	MYLIP	NM_198321.3	ok
223412_at	KBTBD7	NM_001128205.1	ok
226038_at	LONRF1	NM_015000.3	ok
226064_s_at	DGAT2	NM_020733.1	ok
226509_at	ZNF641	NM_001193329.1	ok
226547_at	MYST3	NM_052854.2	ok
226568_at	FAM102B	NM_015444.2	ok
226576_at	ARHGAP26	NM_001101.3	target sequence is downstream of first polyA-site and upstream of other polyA-site(s)
227899_at	VIT	NT_011295.11	ok
228410_at	GAB3	NM_018946.3	target sequence is downstream of first polyA-site and upstream of other polyA-site(s)
229487_at	EBF1	NM_024949.5	ok
229839_at	SCARA5	NM_017918.4	ok
230180_at	---	NM_013352.2	target sequence maps to intron sequence of DDX17 (NM_006386.4)
235306_at	GIMAP8	NM_024657.4	ok
235956_at	KIAA1377	NM_030917.3	target sequence is downstream of first polyA-site and upstream of other polyA-site(s)
236361_at	GALNTL2	NM_001130415.1	ok
237475_x_at	CCDC152	NM_024665.4	ok
239629_at	CFLAR	NM_033161.2	target sequence maps with first oligo to the 3' end of the last exon of NM_001127184.2

242738_s_at	ZFHX3	NM_003781.3	ok
244766_at	LOC100271836 /// LOC100288704 /// LOC440354 /// LOC595101 /// LOC641298 /// SMG1	NM_003255.4	ok
43427_at	ACACB	NM_170746.2	target sequence maps to different genomic areas (LOC…) but not to RNA sequences

Table S1. Controlling of Affymetrix probe set annotation (only for increased genes).

Probeset_ID	Gene Symbol (decreased genes)	RefSeq sequence Release 46	Comment
1558105_a_at	---	NT_079573.4	24477 bp at 5' side: carbohydrate sulfotransferase 76568 bp at 3' side: sodium hydrogen exchanger 7
200014_s_at	HNRNPC	NM_031314.2	ok
200606_at	DSP	NM_004415.2	ok
200654_at	P4HB	NM_000918.3	ok
200663_at	CD63	NM_001780.4	ok
200700_s_at	KDELR2	NM_006854.3	ok
200704_at	LITAF	NM_004862.3	ok
200734_s_at	ARF3	NM_001659.2	ok
200745_s_at	GNB1	NM_002074.3	ok
200757_s_at	CALU	NM_001219.4	ok
200816_s_at	PAFAH1B1	NM_000430.3	ok
200891_s_at	SSR1	NM_003144.3	ok
200902_at	SEP15	NM_004261.3	ok
200989_at	HIF1A	NM_001530.3	ok
200998_s_at	CKAP4	NM_006825.3	ok
201097_s_at	ARF4	NM_001660.3	ok
201099_at	USP9X	NM_001039590.2	target sequence is downstream of first polyA-site and upstream of other polyA-site(s)
201148_s_at	TIMP3	NM_000362.4	ok
201162_at	IGFBP7	NM_001553.1	ok
201261_x_at	BGN	NM_001711.4	ok
201278_at	DAB2	NM_001343.2	ok

201286_at	SDC1	NM_001006946.1	ok
201330_at	RARS	NM_002887.3	ok
201462_at	SCRN1	NM_001145513.1	ok
201470_at	GSTO1	NM_004832.2	ok
201560_at	CLIC4	NM_013943.2	ok
201594_s_at	PPP4R1	NM_001042388.1	ok
201719_s_at	EPB41L2	NM_001199388.1	ok
201994_at	MORF4L2	NM_001142418.1	ok
202052_s_at	RAI14	NM_015577.2	ok
202074_s_at	OPTN	NM_001008211.1	ok
202369_s_at	TRAM2	NM_012288.3	ok
202418_at	YIF1A	NM_020470.2	ok
202591_s_at	SSBP1	NM_003143.1	ok
202619_s_at	PLOD2	NM_182943.2	ok
202669_s_at	EFNB2	NM_004093.3	ok
202693_s_at	STK17A	NM_004760.2	ok
202696_at	OXSR1	NM_005109.2	ok
202722_s_at	GFPT1	NM_002056.2	ok
202986_at	ARNT2	NM_014862.3	ok
203020_at	RABGAP1L	GNOMON 19020193, XM_003118496.1	ok, target sequence is downstream of NM_014857.3
203072_at	MYO1E	NM_004998.2	ok
203083_at	THBS2	NM_003247.2	ok
203201_at	PMM2	NM_000303.2	ok
203349_s_at	ETV5	NM_004454.2	ok
203404_at	ARMCX2	NM_014782.5	ok
203510_at	MET	NM_001127500.1	ok
203620_s_at	FCHSD2	NM_014824.2	ok
203675_at	NUCB2	NM_005013.2	ok
203810_at	DNAJB4	GNOMON 19430213.m, AB209922.1	ok, target sequence overlaps with 3' end of NM_007034.3
203939_at	NT5E	NM_002526.3	ok
204004_at	PAWR	GNOMON 17696263.m	ok, target sequence is 1809 bp downstream of NM_002583.2
204017_at	KDELR3	NM_006855.2	ok
204671_s_at	ANKRD6	NM_014942.3	ok
206298_at	ARHGAP22	NM_021226.2	ok
206580_s_at	EFEMP2	NM_016938.4	ok
207358_x_at	MACF1	NM_012090.4	ok
209094_at	DDAH1	NM_012137.3	ok
209526_s_at	HDGFRP3	NM_016073.2	ok
209578_s_at	POFUT2	NM_015227.4	ok

211071_s_at	MLLT11	NM_006818.3	ok
211202_s_at	KDM5B	NM_006618.3	ok
211325_x_at	LOC171220 (DSTNP2)	NR_033796.1	ok
211725_s_at	BID	NM_197966.1	ok
212256_at	GALNT10	NM_198321.3	ok
212344_at	SULF1	NM_001128205.1	ok
212565_at	STK38L	NM_015000.3	ok
212822_at	HEG1	NM_020733.1	ok
212848_s_at	C9orf3	NM_001193329.1	ok
213059_at	CREB3L1	NM_052854.2	ok
213338_at	TMEM158	NM_015444.2	ok
213867_x_at	ACTB	NM_001101.3	ok
214316_x_at	---	NT_011295.11	207 bp at 5' side: calreticulin precursor; 1743 bp at 3' side: UV excision repair Protein RAD23 homolog A, invers to GNOMON 3910343
218189_s_at	NANS	NM_018946.3	ok
218775_s_at	WWC2	NM_024949.5	target sequence is downstream of first polyA-site and upstream of other polyA-site(s)
218802_at	CCDC109B	NM_017918.4	ok
218854_at	DSE	NM_013352.2	ok
219038_at	MORC4	NM_024657.4	ok
221007_s_at	FIP1L1	NM_030917.3	ok
221031_s_at	APOLD1	NM_001130415.1	ok
222634_s_at	TBL1XR1	NM_024665.4	ok
222978_at	SURF4	NM_033161.2	target sequence is downstream of first polyA-site and upstream of other polyA-site(s)
223374_s_at	B3GALNT1	NM_003781.3	target sequence includes first polyA site
224560_at	TIMP2	NM_003255.4	ok
224677_x_at	C11orf31	NM_170746.2	ok
224771_at	NAV1	NM_020443.4	ok
224955_at	TEAD1	NM_021961.5	ok
225152_at	ZNF622	NM_033414.2	ok
225188_at	RAPH1	NM_213589.1	ok
227280_s_at	CCNYL1	NM_001142300.1	ok
227320_at	FAM101A	NM_181709.4	ok
227566_at	NTM	NM_016522.2	ok
227638_at	KIAA1632 (EPG5)	NM_020964.2	ok
228293_at	DEPDC7	NM_001077242.1	ok
229555_at	GALNT5	NM_014568.1	ok
229582_at	INO80C	NM_001098817.1	ok

231841_s_at	KIAA1462	NM_020848.2	ok
231999_at	ANKRD11	NM_013275.4	ok
243864_at	CCDC80	GNOMON 6299293	ok, oligo #11 not matching to variant 1(NM_199511.1) and 2 (NM_199511.1), first polyA site upstream, second downstream

Table S2. Controlling of Affymetrix probe set annotation (only for decreased genes).
